# Viroid ecology in hops (*Humulus lupulus* L.): high prevalence in commercial systems but low presence in wild populations

**DOI:** 10.3389/fmicb.2025.1652923

**Published:** 2026-01-05

**Authors:** Swati Jagani, Christina Krönauer, Ute Born, Michael Helmut Hagemann

**Affiliations:** 1 Department of Production Systems of Horticultural Crops, University of Hohenheim, Stuttgart, Germany; 2 Bavarian State Research Center for Agriculture, Institute for Crop Science and Plant Breeding, Wolnzach, Germany

**Keywords:** Cocadviroid, Hostuviroid, Carlavirus, *Humulus lupulus* L., Sanger sequencing, RT-PCR

## Abstract

**Introduction:**

Hop (*Humulus lupulus* L.), a vital crop in the brewing industry, is increasingly threatened by infections caused by viroids and viruses. The extensive use of vegetative propagation in hop cultivation facilitates the accumulation and dissemination of these pathogens. However, little is known about their prevalence and ecological behavior in non-commercial settings. This study provides a comprehensive overview of viroid and virus infections across Germany, with particular attention to their occurrence and potential transmission across commercial, settlement, and wild hop populations.

**Methods:**

Between 2020 and 2023, 418 hop leaf samples from commercial (*n* = 345), settlement (*n* = 29), and wild (*n* = 44) populations were collected. Viroid and virus detection was performed using RT-PCR and PCR. To investigate possible cross-species transmission and sequence variation, HSVd-positive samples from hops and nearby grapevines were further analyzed via Sanger sequencing.

**Results:**

Viroid screening revealed that the citrus bark cracking viroid (CBCVd; *Cocadviroid rimocitri*) was confined to commercial hop cultivation. This study also marks the first confirmed detection of hop stunt viroid (HSVd; *Hostuviroid impedihumuli*) in commercial hop fields in Germany. Virus screening showed that hop latent virus (HpLV; *Carlavirus latenshumuli*) and american hop latent virus (AHpLV; *Carlavirus americanense*) were exclusively found in commercial hops. Hop mosaic virus (HpMV; *Carlavirus humuli*) was detected across all three groups—commercial, settlement, and wild populations. Arabis mosaic virus (ArMV; *Nepovirus arabis*) and apple mosaic virus (ApMV; *Ilarvirus ApMV*) were identified in both commercial and wild hops but were absent from settlement samples. Overall, commercial hop populations exhibited the highest pathogen burden, frequently harboring multiple viroid and virus infections. These findings underscore the importance of using certified, pathogen-free planting material, implementing early detection strategies, and updating plant passport regulations to include high-risk pathogens. While prevalence estimates reflect risk-based sampling from key production regions, the study provides a solid basis for enhancing pathogen surveillance and improving preventive measures in hop cultivation.

## 1 Introduction

Hop (*Humulus lupulus* L.) is a high-value crop cultivated worldwide, primarily for its use in beer production. Germany is one of the world’s leading hop producers, accounting for approximately 35% of global hop production in 2023, with a total yield of 41,234 tonnes out of the global 118,415 tonnes (BarthHaas, 2024). It plays a major role in the global specialty hop market, offering both aroma and bittering varieties tailored to diverse brewing styles (Pavlovič et al., 2012; Šrédl et al., 2020). Hop cultivation is threatened by a wide range of pathogens, including fungi, oomycetes, viruses, and viroids, which can be transmitted through sap, mechanical injury, or insect vectors (Gent and Ocamb, 2009; Pethybridge et al., 2008). Among these, viruses and viroids represent a unique class of systemic pathogens that rely entirely on host cellular machinery for replication and movement. Viruses are composed of a nucleic acid genome (DNA or RNA) enclosed in a protein coat, whereas viroids are smaller, circular, single-stranded RNAs that lack protein-coding capacity and a protective capsid (Flores et al., 2011; Adkar-Purushothama and Perreault, 2020). To date, five viroids have been reported in hops: citrus bark cracking viroid (CBCVd; *Cocadviroid rimocitri*) (Jakse et al., 2015), hop latent viroid (HLVd; *Cocadviroid latenshumuli*) (Patzak et al., 2021), hop stunt viroid (HSVd; *Hostuviroid impedihumuli*) (Sasaki and Shikata, 1977; Sano et al., 2001), *apple fruit crinkle viroid* (AFCVd) (Sano et al., 2004), and citrus exocortis viroid (CEVd; *Pospiviroid exocortiscitri*) (Hagemann et al., 2023). The primary mode of transmission of hop viroids is through mechanical injuries caused by agricultural machinery and during vegetative propagation (Hadidi et al., 2022). Among these, CBCVd belonging to the genus *Cocadviroid* exhibits the highest pathogenicity in hops. Infected hop plants exhibit severe stunting, leaf curling, and stem bark cracking, often leading to plant death within three to five years (Jakse et al., 2015; Štajner et al., 2019). The complete 284 bp genome of CBCVd was first sequenced from a dwarf grapefruit (*Citrus paradisi*) in Israel in 1991 (Puchta et al., 1991). CBCVd was later identified in hop plants in Slovenia in 2015 and subsequently detected in hops in Germany in 2019 (Jakse et al., 2015; Julius Kühn-Institut, 2019). HLVd belongs to the genus *Cocadviroid*. It is the most prevalent viroid in hop cultivation worldwide and is typically considered latent, as it does not induce visible phenotypic disease symptoms. Even without outward signs, HLVd infection may reduce bitter acid levels and alter terpene composition, negatively affecting aroma and brewing quality (Puchta et al., 1988a; Patzak et al., 2021; Štajner et al., 2019). A study by Patzak et al. (2021) reported reductions in *α*-bitter acid levels by 8.8 to 34% and xanthohumol content by 3.9 to 23.5% in the cultivar ‘Saaz’. More recently, HLVd has also been identified as a major pathogen in cannabis, causing stunting, reduced trichome production, and losses of up to 50% in THC and terpene content, posing a substantial economic threat (Adkar-Purushothama et al., 2023). HSVd is classified under *Hostuviroid*. It is less widespread in hops but poses a notable threat due to its cross-species transmission. Phylogenetic analyses by Sano et al. (2001) revealed that HSVd-hop isolates cluster closely with grapevine-derived HSVd-g subtype 1, indicating that hop-infecting variants likely originated from grapevine populations. Long-term experimental infections demonstrated that HSVd-g variants originating from grapevine can adapt when maintained in hops. These adapted variants developed the same mutations as those found in naturally occurring HSVd strains during hop epidemics (Kawaguchi-Ito et al., 2009). In hops, HSVd symptoms vary depending on cultivar. Kappagantu et al. (2017) reported a 73% reduction in side-arm length and 29 and 26% decreases in internode and shoot length, respectively, in infected ‘Willamette’ plants. Over five years, *α*-acid and *β*-acid contents in ‘Willamette’ cones declined by 37 and 36%, respectively. Similar symptoms, including moderate epinasty and delayed climbing, were observed in the cultivar ‘Glacier’ (Eastwell and Nelson, 2007).

In contrast, AFCVd from genus *Apscaviroid* has so far been detected only in limited regions, specifically parts of Japan, China, and the USA, indicating that its distribution remains geographically restricted (EPPO, 2024). Lastly, CEVd, a member of the genus *Pospiviroid*, which has not been reported to cause natural infections in hops but has been shown to do so under experimental conditions (Hagemann et al., 2023). According to Puchta et al. (1991), it shares part of its sequence with CBCVd, suggesting a possible evolutionary relationship and risk of outbreak. However, given the limited relevance of AFCVd and CEVd to natural infections in German hops, these viroids were not included in our screening. Following the viroids, several viruses have also been reported in hops, with 16 identified to date (Gargani et al., 2017). The most prevalent viruses worldwide include hop latent virus (HpLV; *Carlavirus latenshumuli*), american hop latent virus (AHpLV; *Carlavirus americanense*), and *hop mosaic virus* (HpMV; *Carlavirus humuli*), all belonging to the genus *Carlavirus*; arabis mosaic virus (ArMV; *Nepovirus arabis*), classified under the genus *Nepovirus*; and apple mosaic virus (ApMV; *Ilarvirus ApMV*), a member of the genus *Ilarvirus*.

HpLV, AHpLV, and HpMV are primarily transmitted by the hop-damson aphid (*Phorodon humuli*) in a non-persistent manner. Additional aphid species, such as *Myzus persicae*, and mechanical transmission also aid in their spread (Pethybridge et al., 2008; Crowle et al., 2006; Ziegler et al., 2014). HpLV is globally distributed and appears to be symptomless in most cultivars (Pethybridge et al., 2008). However, in cultivars such as ‘Agate’, yield losses of up to 70% and a 44% reduction in alpha acid content have been observed in the first year (Pethybridge et al., 2004).

AHpLV occurs frequently in mixed infections with HpMV and HpLV. It remains phenotypically inconspicuous yet reduces cone yield by 14% and alpha acids by 12% in cultivars like ‘Chinook’ (Eastwell and Druffel, 2012; Probasco and Murphey, 1996). HpMV infections are largely cultivar dependent. While most modern cultivars remain asymptomatic, sensitive Goldings-type cultivars show clear symptoms such as chlorotic vein-banding, leaf curling, stunting, and yield loss. In some cases, infected plants die prematurely (Hataya et al., 2001; Pethybridge et al., 2008).

Among viruses affecting hop production, ArMV is particularly notable for its role in complex diseases such as nettle head, bare-bine, and split leaf blotch. Transmission occurs via soil-borne dagger nematodes (*Xiphinema diversicaudatum*), which acquire the virus by feeding on infected roots and transmitting it during migration (Jha and Posnette, 1961; Pethybridge et al., 2008). Nettlehead disease, evident in early summer, includes leaf mottling, vein clearing, enations on midribs and main veins, upward rolling, and shoot failure. Affected plants are severely stunted and produce few cones (Thresh et al., 1972). In a two-year survey on the cultivar ‘Bullion’, Thompson and Neve (1971) reported dry cone yield reductions of 26 and 23% in 1969 and 1970, respectively. Alpha acid content dropped by 7 and 8% in the same years.

ApMV can reduce hop yield and brewing quality, as shown in experimental infections of the ‘Saaz’ cultivar (Matsui et al., 2017). It is mainly transmitted through vegetative propagation and mechanical injury via sap (Grimová et al., 2016). Symptoms include chlorotic ring spots, necrosis, and oak-leaf line patterns (Pethybridge et al., 2008). In 2011, ‘Saaz’ hop plants infected with ApMV produced only 0.9 t/ha as compared to the uninfected hops which yielded around 2.0 t/ha and also showed reduced humulone content (4% vs. 5%) (Matsui et al., 2017).

As dioecious species, hops are propagated clonally via rhizomes or cuttings to preserve desirable female traits. This practice, however, can increase the risk of disease transmission. For example, field trials by Lombard et al. (2014) showed that plants established from non-certified rhizomes exhibited high infection rates with apple mosaic virus and american hop latent virus, while certified stock remained virus-free. These findings highlight the importance of using clean planting material to prevent the introduction and accumulation of viral pathogens in hop yards.

Beyond propagation practices, understanding how viruses and viroids persist in the environment is crucial for effective disease management. Wild hop populations, often growing near cultivated fields, may play a role in maintaining these pathogens due to their capacity to harbor infections asymptomatically and their proximity to cultivated hops. Evidence from Western Siberia supports this, with wild hops exhibiting a high virus burden (95.5% overall infection; HpLV 24.5%, ApMV 8.2%, ArMV 5.5%) (Khlebova et al., 2024). A separate study examining viroid presence in wild hops from Southern Italy found no HSVd, even though the viroid was detected in neighboring fruit crops (Ragozzino et al., 2008). These region-specific patterns underscore the complexity of pathogen distribution in unmanaged hops and highlight the need to further investigate their epidemiological significance in long-term disease ecology.

This study systematically examines hop plants from three distinct provenance types in Germany, commercial, settlement, and wild, using molecular diagnostics to detect virus and viroid infections. Commercial hops are intensively cultivated in managed fields; settlement hops are semi-managed plants located near human dwellings; and wild hops grow in unmanaged natural habitats, like forests. The study objectives are to: (1) assess the prevalence and distribution of key hop-infecting viroids (CBCVd, HLVd, HSVd), with a particular focus on comparing wild and commercial populations; (2) compare the sequence variation of HSVd isolates from hop and grapevine hosts to identify potential host adaptations; and (3) examine the prevalence and host range of major hop viruses (HpLV, AHpLV, HpMV, ArMV, ApMV) across all three hop population types.

## 2 Materials and methods

### 2.1 Sample collection

Hop leaf samples were collected from three different provenances across Germany between 2020 and 2023. The samples were classified as commercial, settlement, or wild, depending on cultivation context (Data sheet 2). Commercial samples were obtained from intensively managed hop fields used for agricultural production and were considered high-risk areas due to previous pathogen detections. Settlement samples were collected from plants growing in residential areas, such as private gardens and roadsides. Wild samples were gathered from unmanaged habitats where hops grow naturally. Both were classified as low risk due to the absence of reported viroid outbreaks. Wild and settlement samples were collected over five consecutive days, with hop locations identified using the Pl@ntNet™ app (CIRAD, Montpellier, France). For each plant, three to seven leaves were placed in press-seal plastic bags, stored on ice, and transported to the laboratory for viroid analysis. Additional details on sampling locations are provided in Data sheet 2.

Grapevine leaf samples were gathered in 2024 from two sources: (i) wild grapevines growing near HSVd-positive hop plants, and (ii) a commercial vineyard located adjacent to hop production fields. Similar to hop sampling, three to seven leaves per plant were collected and stored in press-seal plastic bags. Samples were kept on ice during transport and subsequently frozen at −80 °C until RNA extraction.

### 2.2 CBCVd-monitoring sample collection and analysis

Samples for CBCVd detection were collected in Bavaria as part of the CBCVd monitoring program conducted by the Bayerische Landesanstalt für Landwirtschaft, Freising, Germany. Sampling was focused on high-risk plots, with sites selected based on their likelihood of CBCVd infection. Fields near known infection sites or those showing signs of disease in aerial images were included in the monitoring. Hop gardens were inspected for symptomatic plants based on farmer reports, by visual evaluation, and by aerial photos captured using a drone (DJI Mavic 2 Pro). Symptoms to look out for were signs of stunted growth or leaf deformation. Ten leaves were collected per sample and stored in press-seal plastic bags under cool conditions until further analysis. Plant coordinates were recorded using a GIS application to enable future re-sampling. For CBCVd analysis, samples were sent to Bodengesundheitsdienst GmbH, Ochsenfurt, Germany where the analysis was performed following Seigner et al. (2020).

### 2.3 RNA extraction

Hop or grapevine leaf samples (section 2.1) were flash-frozen and ground in liquid nitrogen. A total of 100 mg of the homogenized material was used for RNA extraction with the Monarch® Total RNA Miniprep Kit (New England Biolabs, Ipswich, USA), following the manufacturer’s protocol. The quality and concentration of the extracted RNA were assessed using a NanoDrop™ 1,000 spectrophotometer (Thermo Fisher, Waltham, USA).

### 2.4 PCR analysis of viroids and viruses

A total of 500 ng purified RNA from hop leaf samples was reverse transcribed into cDNA in a 10 μL reaction volume using the LunaScript® cDNA Synthesis Kit (New England Biolabs, USA), following the manufacturer’s instructions. While viroids were individually tested, cDNA extracts were pooled within each provenance group prior to PCR analysis for virus detection. The rationale and limitations of the pooling approach are described in detail in the Data sheet 3. PCR analysis was conducted with Q5® High-Fidelity DNA Polymerase (New England Biolabs, Ipswich, USA). Each reaction was performed in a 20 μL total volume, containing 1 μL of cDNA as the template and 0.5 μM of forward and reverse primers, following the kit’s standard protocol. Primer details for the detection of HLVd, HSVd, AHpLV, ArMV, HpLV, HpMV, and ApMV are provided in Data sheet 4. Primer optimization details, including concentrations and BLAST verification, are summarized in Table 1 (Supplementary data).

**Table 1:** Number of samples tested for hop viroids in commercial, settlement, and wild hop populations in Germany.

Provenance	Total samples tested	CBCVd	HLVd	HSVd
Commercial	345	256	320	5
Settlement	29	0	1	0
Wild	44	0	3	0
**Grand total**	**418**	**256**	324	5

The table shows the total number of samples tested and the number of positive detections for CBCVd, HLVd and HSVd for each provenance.

For the visualization and confirmation of PCR amplification, agarose gel electrophoresis was performed using a 1% gel prepared by dissolving 1 g of Bioproducts SeaKem® LE agarose (Rockland, USA) in 100 mL of TBE buffer. For band visualization, 5 μL of peqGREEN DNA/RNA Dye (Peqlab by VWR, Darmstadt, Germany) was added before pouring the gel. 5 μL of PCR products were mixed with 1 μL of 6 × TriTrack loading dye (Thermo Fisher Scientific™, Waltham, USA) and loaded onto the gel. A 100 bp Quick-Load DNA ladder (7 μL, New England Biolabs, USA) was used for fragment size determination. Electrophoresis was run at 100 V for 50–60 min. Example gel images showing PCR amplification results for viroid and virus detection are provided in Presentation 1 (Supplementary data).

### 2.5 HSVd sequencing and analysis

To characterize HSVd variants, RT-PCR amplicons from HSVd-positive hop and commercial grapevine samples were subjected to Sanger sequencing. Prior to sequencing, PCR products were purified using the Exo-CIP Rapid PCR Cleanup Kit (New England Biolabs, Ipswich, USA). Each reaction consisted of 5 μL PCR product, 3 μL Exo-CIP A, 3 μL Exo-CIP B, and 10 μL deionized water. The samples were then submitted to Macrogen Europe (Amsterdam, Netherlands) for Sanger sequencing.

To obtain sequence data, raw Sanger reads were imported into Geneious Prime (Biomatters Ltd., Auckland, New Zealand) and trimmed using an error probability threshold of 0.01 (corresponding to a 1% error rate). Primer sequences were removed, allowing up to two mismatches and requiring a minimum match length of five base pairs. The trimmed sequences were then mapped to the HSVd reference genome (GenBank accession no. NC_001351) using the Geneious Mapper with the “highest sensitivity” setting and five iterations. The assembly resulted in a consensus sequence based on highest quality (60%), excluding the reference sequence. To ensure full-length coverage and minimize data loss at primer sites, we used overlapping primers targeting conserved HSVd regions beyond the upper conserved and lower conserved region (Data sheet 4).

## 3 Results

### 3.1 Viroid screening

To evaluate viroid prevalence across different hop growing provenances, 418 individual leaf samples were tested for CBCVd, HLVd, and HSVd. Samples were characterized into commercial (*n* = 345), settlement (*n* = 29), and wild (*n* = 44) populations, as described above. All commercial samples originated from designated high-risk areas with known CBCVd presence, while settlement and wild hops represented low-risk regions. CBCVd was detected exclusively in 256 (74%) commercial samples, confirming its confinement to high-risk cultivation regions. HLVd was the most widespread viroid, occurring in 320 (93%) commercial samples and, notably, also detected in one settlement sample and three wild samples. HSVd was detected in five commercial samples and was absent from both settlement and wild populations as summarized in Table 1.

### 3.2 Virus screening

To broaden the scope of pathogen prevalence, we extended the study to include virus screening. A total of same 418 hop samples were combined into 45 pools and tested by PCR for the presence of five viruses: HpLV, AHpLV, HpMV, ArMV, and ApMV (Table 2). Samples were grouped into pools of five to ten individual plants based on the three hop growing provenances: commercial (*n* = 37), settlement (*n* = 3), and wild (*n* = 5). Pooling was performed to obtain a general overview of virus prevalence across these cultivation systems and to increase testing efficiency.

**Table 2:** Number of samples tested for hop viruses in commercial, settlement, and wild hop populations in Germany.

Provenance	Total pools tested	HpLV	AHpLV	HpMV	ArMV	ApMV
Commercial	37	29	12	33	4	37
Settlement	3	0	0	1	0	0
Wild	5	0	0	1	1	2
**Grand total**	**45**	**29**	**12**	**35**	**5**	**39**

The table shows the total number of pools tested and the number of positive detections for HpLV, AHpLV, HpMV, ArMV, and ApMV for each provenance.

HpLV and AHpLV were detected exclusively in commercial pools, with 29 (78%) and 12 (32%) positive detections, respectively. HpMV notably had the widest distribution, identified in 33 (89%) commercial pools, as well as in one settlement and one wild pool. ArMV was found in four commercial pools and one wild pool, while no detections were observed in settlement areas. Finally, ApMV was detected in all 37 (100%) commercial pools and in two wild pools, while no settlement pools tested positive. Example amplicon sequences for new primer pairs are provided in Data sheet 5.

### 3.3 HSVd sequence variation between hop and grapevine

**Figure 1:**
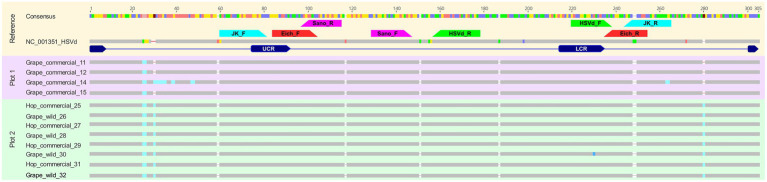
Sanger sequencing alignment of HSVd-positive samples from hop and grapevine. Consensus sequences were generated from HSVd-positive PCR products derived from commercial hop fields, nearby commercial grapevine fields, and wild grapevines growing near hop farms. Sequences were aligned to the HSVd reference genome (NC_001351). Colored bars represent nucleotide positions aligned with the consensus sequence. Annotated regions indicate primer binding sites (JK, Eich, Sano, and HSVd primer sets, as described in Data sheet 4), as well as known HSVd functional domains, including the upper conserved region (UCR) and lower conserved region (LCR).

HSVd-positive samples from commercial hop plants and wild grapevines growing in proximity as well as samples from a commercial grapevine plantation were selected for Sanger sequencing to investigate sequence variability across host species. HSVd was detected in all three sources, but not in all samples. Sequence analysis showed that HSVd isolates were highly conserved overall, with all consensus sequences displaying high similarity to the reference genome (NC_001351) mostly of 94%, except the accession ‘Grape_commercial_14’ with 91% pairwise identity, respectively (Figure 1). Even within the accessions from the commercial grapevine plantation these accessions differ by 7 nucleotides. In the following we describe only the three major differences within our samples allowing to clearly separate commercial grapevines from commercial hops and wild grapevines; first, the commercial grapevines have TC/TA dinucleotide, whereas it is substituted by a GA for all commercial hop and wild grapevine accessions at positions 25–26 (TC/TA → GA). Second, the commercial grapevines except ‘Grape_commercial_14’, show a single-nucleotide deletion at position 30 (C). Third, the commercial grapevines showed another deletion at position 280 (G), where the commercial hops and wild grape have a guanine (G).

## 4 Discussion

This study investigates the prevalence and distribution of viroids and viruses in German hop cultivation, focusing on their occurrence across commercial, settlement, and wild systems. HLVd was the most frequently detected viroid, found in all three provenances, confirming its widespread distribution across all production systems. It often escapes visual detection, but its presence lowers alpha acid content in affected cultivars, such as ‘Saaz’, compromising brewing (Patzak et al., 2021). Although HLVd was detected in wild and settlement hops populations, its presence is unlikely to result from seed or pollen transmission. Matoušek et al. (2008) demonstrated that HLVd is efficiently degraded during pollen maturation, preventing generative transfer. The spread in settlement areas may be explained by vegetative propagation using infected nursery stock or shared cuttings. In wild populations, the viroid may have spread through human-mediated routes, including dispersed rhizomes, use of contaminated tools, or disposal of gardening waste from nearby cultivated areas. CBCVd was detected in 74% of commercial samples, mostly derived from the CBCVd-infected region in the Hallertau, but was absent from both wild and settlement populations. This limited distribution supports previous reports of local spread of CBCVd, which occurs primarily via mechanical transmission (Jakse et al., 2015). It should be noted that prevalence estimates are influenced by risk-based sampling, particularly in commercial settings where CBCVd monitoring targeted high-risk plots within key hop-growing regions. The results indicate a locally contained CBCVd outbreak within a specific area of the Hallertau region, while, to the best of our knowledge, the majority of the Hallertau and other German hop production areas remain CBCVd-free. This observation is consistent with results from additional monitoring activities conducted by regional plant protection services (*Pflanzenschutzdienste der Länder*), including the State Institute for Agriculture, Nutrition and Rural Areas Baden-Württemberg (LTZ Augustenberg, personal communication). Our findings reflect likely pathogen presence within intensively monitored hotspots rather than a randomized nationwide distribution.

However, regarding another hop pathogenic viroid, HSVd, to the best of our knowledge, this study represents the first report of HSVd in five commercial hop samples from Germany. Certainly, HSVd has been found earlier in grapevine (Puchta et al., 1988b), it has not been found to interfere with hop production. Previous research by Seigner et al. (2014), based on viroid and virus monitoring conducted between 2008 and 2013 in Germany, reported HSVd in nine plants from the Huell germplasm collection, including five ‘Horizon’ plants imported from the USA in 2001 and four neighboring cultivars. The infection was confined to the germplasm collection, with no detections in commercial hop fields. In our study, HSVd was identified in hop fields located adjacent to wild grapevines, a known HSVd host that often remains asymptomatic and may serve as a silent reservoir in mixed cropping systems (Sano et al., 2001). This geographical association suggests the potential for cross-species transmission between grapevines and hops, indicating possible epidemiological links. Phylogenetic studies have shown that HSVd variants from grapevine, particularly the HSVd-g subtype 1, cluster closely with hop-derived isolates, suggesting grapevine as a likely original source of hop-infecting strains (Sano et al., 2001; Kawaguchi-Ito et al., 2009). Over time, these grapevine-derived HSVd variants have accumulated adaptive mutations that enable them to persist in hop hosts, where they become pathogenic and lead to crop deterioration in both yield and quality (Kappagantu et al., 2017; Eastwell and Nelson, 2007).

To further investigate the origin and host adaptation of HSVd in hops, we analyzed full-length sequences from hop, commercial grapevine, and wild grapevine samples (Figure 1). The resulting alignment showed high similarity among isolates and with the HSVd reference genome (NC_001351), supporting strong sequence conservation across hosts. Interestingly, three minor nucleotide changes were identified exclusively in the commercial grapevine samples. While limited in number and unlikely to affect viroid function, their restricted occurrence may indicate early-stage host-specific divergence. These observations are consistent with the gradual accumulation of mutations described by Kawaguchi-Ito et al. (2009), who reported five adaptive changes in HSVd genomes following 10–15 years of persistence in hop plants. In our case, the low level of variation may represent an earlier phase of cross-host transmission, where insufficient time has passed for substantial adaptation to occur. Alternatively, the HSVd variant present may already be broadly compatible with both hop and grapevine, requiring little additional sequence modification to establish infection.

Expanding on viroid distribution, we examined virus prevalence across hop systems by examining pools made from the viroid monitoring. Virus incidence mirrored viroid patterns, peaking in commercial hops. Both HpLV and AHpLV were exclusively detected in commercial hops at 78 and 32%, respectively. The presence of viruses in commercial settings has been previously reported; for example, Hay et al. (1992) showed that HpLV and AHpLV were widespread in New Zealand’s commercial hop cultivars, often persisting as latent infections. The authors speculated that these viruses may spread via mechanical transmission, natural root grafts, or propagation from infected planting material.

ArMV was found in four commercial pools and one wild pool. Although rare, this detection is concerning. ArMV infects over 90 plant species across various families, with major hosts including grapevine and raspberry (EPPO, 2025; CABI, 2022). It is transmitted by soil-borne nematodes and can remain undetected to the naked eye (Jha and Posnette, 1961; Pethybridge et al., 2008). Given its pathogenicity in hops, ArMV should be included in future monitoring and plant passport frameworks. Based on commercially sourced hop plants we received, certified planting material is indeed routinely tested for HpMV, ArMV, HSVd, and CBCVd, as well as for Verticillium wilt—going beyond the standard EU plant passport requirements. Despite this, both HpMV and ApMV were still detected in commercial hop samples. However, HpMV was also present in settlement and wild hop populations, whereas ApMV appeared only in wild populations. A similar trend was observed by Khlebova et al. (2024) in wild hops from Western Siberia, where ApMV, HpLV, and ArMV were found in natural populations. According to the authors, these findings highlight wild hops as potential virus reservoirs capable of transmitting pathogens to nearby cultivated varieties through aphid vectors or mechanical contact.

The consistently high pathogen load in commercial hops can be attributed to vegetative propagation and monoculture. Clonal propagation via rhizomes or cuttings ensures uniformity but facilitates pathogen build-up, while consecutive monoculture cycles alter root exudates and soil microbiomes in ways that promote viral persistence. Wu et al. (2022) showed in *Radix pseudostellariae* that such practices reduced microbial diversity, shifted soil viral communities, and promoted accumulation of plant viruses in crop roots. While demonstrated in another crop, these mechanisms are relevant to perennial and vegetatively propagated crops such as hops. Supporting this, Pasha et al. (2025) investigated virus and viroid diversity in German hops using high-throughput sequencing, focusing on pooled leaf material from commercial hop yards across the Hallertau, Tettnang, and Elbe-Saale regions. Their study provided a valuable overview of major pathogens, confirming the widespread presence of HpLV, HpMV, ApMV, HLVd, and the localized occurrence of CBCVd in Hallertau. However, the study was restricted to commercial hop yards, limiting resolution regarding infection dynamics across different hop provenances. In contrast, our study not only encompassed commercial production systems but also included settlement and wild hop populations, thereby revealing distinct ecological patterns and barriers to pathogen persistence. We also report the first occurrence of HSVd in German commercial hops and provide sequence-based evidence linking its presence to neighboring grapevines as a potential cross-host reservoir. By integrating epidemiological context, host biology, and environmental interactions, our findings extend beyond pathogen detection to explain mechanisms of distribution, adaptation, and future risks. Thus, while Pasha et al. (2025) offered an important baseline for the German hop virome, our work extended the understanding of viroid ecology in hops and delivers actionable insights for disease management and climate-resilient production.

In sharp contrast to the high pathogen prevalence in commercial systems, wild hops reproduce sexually through seed and pollen, a process that disrupts the vertical transmission of many systemic pathogens. For example, Matoušek et al. (2008) demonstrated that HLVd, though detectable in early pollen development, is degraded during maturation. During this process, nucleases like HBN1 become active and degrade HLVd RNA. The resulting RNA fragments are larger than siRNAs, indicating that the mechanism is independent of RNA silencing. By the time pollen germinates, HLVd is no longer detectable. Similarly, Steinbachová et al. (2021) reported that viroids such as CBCVd and AFCVd, although abundant in immature anthers, were reduced by approximately 3,600-fold and 800-fold, respectively, in mature pollen of Nicotiana benthamiana compared to leaves. This elimination was attributed to a combination of viroid degradation and suppressed replication during male gametophyte development, rather than general pollen pathogenesis, as mature pollen remained viable and functional. These findings suggest the presence of targeted mechanisms that effectively block vertical transmission during pollen maturation. Consequently, wild hops grown from seed rarely retain systemic pathogens. In contrast, pathogen presence in settlement hops may depend on plant origin, naturally seeded plants tend to be pathogen-free, whereas those introduced from nurseries often carry infections due to vegetative propagation and contaminated source material.

A deeper understanding of these distribution patterns highlights the importance of examining how hop plants defend against RNA-based pathogens such as viroids and viruses. RNA interference (RNAi) is a key plant immune mechanism that utilizes small RNAs, including siRNAs and miRNAs, to target and degrade foreign RNA molecules (Akbar et al., 2022). During infection, viroid-derived small RNAs (vd-sRNAs) are processed by DICER-like enzymes and incorporated into ARGONAUTE, forming the RNA-induced silencing complex that mediates pathogenic RNA silencing (Navarro et al., 2009). Members of the family *Pospiviroidae*, such as PSTVd, replicate in the nucleus by recruiting the host’s DNA-dependent RNA polymerase II to transcribe their RNA genomes (Flores et al., 2005). These RNA-based defense and replication mechanisms are likely to operate similarly in hops.

Environmental factors, particularly temperature, can significantly influence both RNAi efficiency and infection dynamics. High temperatures enhance RNAi by increasing siRNA accumulation and strengthening antiviral defense responses (Szittya et al., 2003). However, elevated temperatures simultaneously accelerate viral replication and systemic spread, leading to increased viral RNA levels and more severe disease symptoms (Ramesh et al., 2021). This dual effect suggests that while RNAi may be more active under higher temperatures, the associated increase in viral load, especially under mixed infection scenarios, may surpass the plant’s silencing capacity and intensify symptom development.

**Figure 2:**
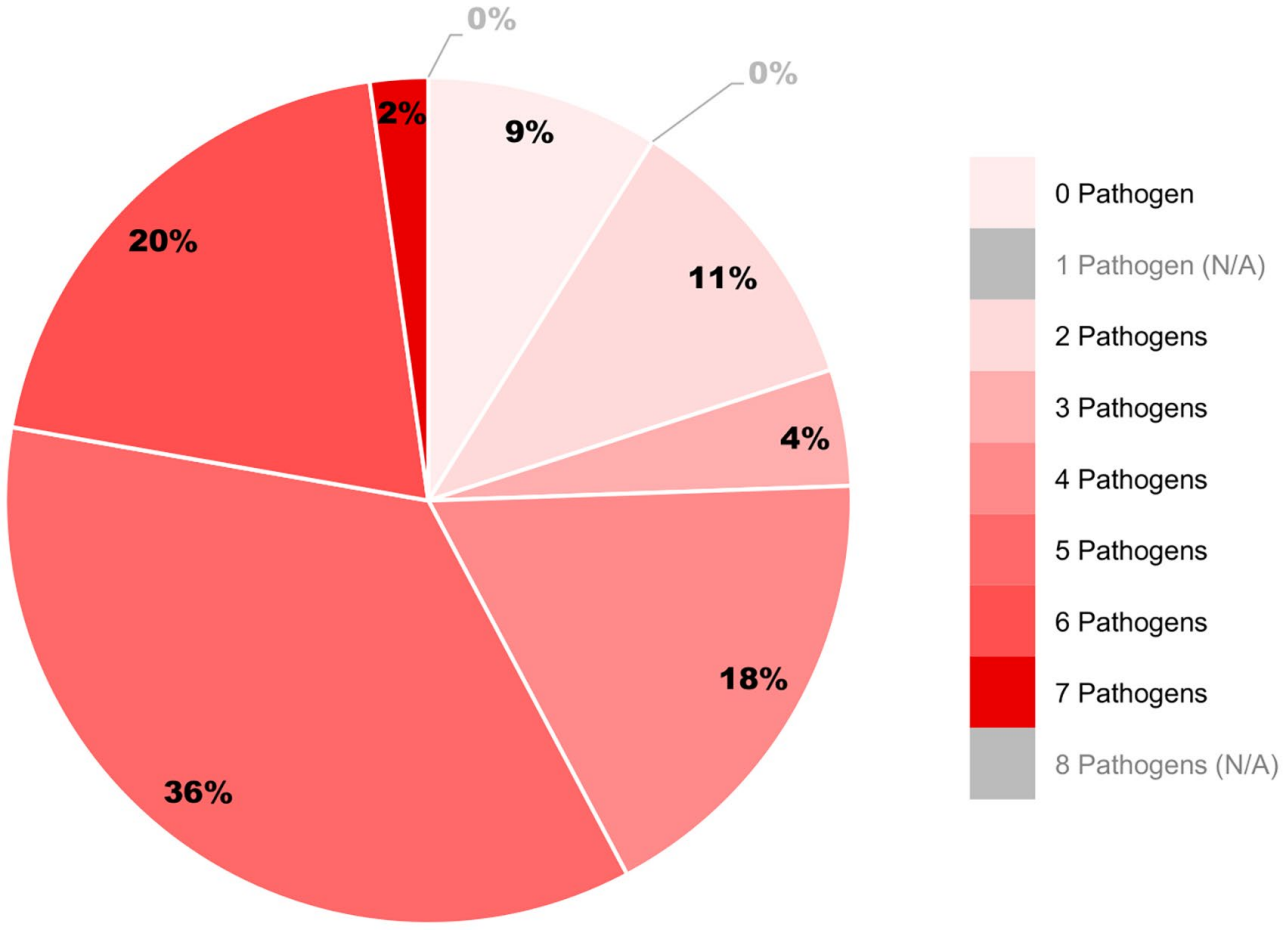
Graph illustrates the distribution of the number of pathogens detected per sample pool, highlighting the prevalence of mixed infections. The analysis includes eight plant pathogens: five viruses and three viroids. Color intensity corresponds to the number of pathogens, ranging from light red (0 pathogens) to deep red (7 pathogens). Each slice is labelled with the percentage of pools in the respective category. Categories with no detection are shown in gray as not applicable (N/A).

Our findings support this concern, as Figure 2 demonstrates that 76% of hop sample pools harbored more than three pathogens, with 2% of samples exhibiting seven distinct infections per sample. This high prevalence indicates that hops already operate under substantial pathogenic pressure, with persistent mixed infections of viroids and viruses in individual plants.

Taken together, these findings suggest that with projected temperature increases due to climate change, the replication and systemic spread of viral and viroid pathogens in hops may intensify. As a result, viral diseases are likely to become more visible and agronomically impactful under future climatic conditions, particularly in commercial systems prone to co-infections. The detection of HSVd in Germany, alongside widespread viroids and viruses, highlights growing risks in commercial systems. Mixed infections, frequently observed, can alter symptoms and complicate diagnostics. To mitigate this, integrated disease management is essential. Certified, pathogen-free planting material remains a cornerstone of prevention in clonally propagated crops (Hadidi et al., 2022). Economic analyses show even modest HSVd-induced yield losses justify the use of clean stock (Davis et al., 2021). Regular updates to monitoring lists, rapid diagnostics, and strict nursery hygiene are critical for early intervention.

This study enhances our understanding of viroid ecology in hops by showing how cultivation practices, propagation methods, and host biology shape pathogen distribution. We demonstrate that human-managed systems bypass natural barriers that restrict systemic pathogen spread. In contrast, wild populations act as ecological filters, limiting pathogen persistence across generations. These insights can inform more resilient production strategies and highlight the need for ongoing surveillance as viroid threats evolve.

## Data Availability

The datasets presented in this study can be found in online repositories. The names of the repository/repositories and accession number(s) can be found in the article/Supplementary material.
