# Supplementary material for: Viroid ecology in hops (Humulus lupulus L.): high prevalence in commercial systems but low presence in wild populations
Source: Front Microbiol. 2026 Jan 5;16:1652923. doi: 10.3389/fmicb.2025.1652923 (PMC12813154; doi:10.3389/fmicb.2025.1652923)
Supplement: Supplementary file 1 [file Data_Sheet_1.docx]

**Viroid Ecology in Hops (Humulus lupulus L): High Prevalence in Commercial Systems but Low Presence in Wild Populations**

## **Authors**

**Swati Jagani ^1^, Christina Krönauer ^2^, Ute Born ^1^, Michael Helmut Hagemann ^1^**

^1^ University of Hohenheim, Production Systems of Horticultural Crops, Emil-Wolff-Str. 25, 70599 Stuttgart, Germany

^2^ Bayerische Landesanstalt für Landwirtschaft, Institute for Crop Science and Plant Breeding, Huell 5 1/3, 85283 Wolnzach

**Data sheet 1: Supplemental File Overview**

| **Item** | **Description** | **Format** | **Content** |
| --- | --- | --- | --- |
| **Data Sheet 2**  **Data Sheet 2_Hop growing systems** | Overview of hop growing systems used in the study (commercial, settlement, and wild populations) | Word -Figures | 12 example pictures of hop plants growing in commercial, settlement and wild |
| **Data Sheet 3**  **Data Sheet 3_Sample pooling rationale** | Explanation of individual testing for viroids and sample pooling strategy for virus detection | Word- Text | Justifies individual testing for viroids (CBCVd, HLVd, HSVd) and pooling for virus detection (HpLV, AHpLV, HpMV, ArMV, ApMV), including rationale, and pooling group size logic. |
| **Data Sheet 4**  **Data Sheet 4_Primers** | Primer and probe sequences used for viroid and virus detection | Word table | Lists all primers and probes by pathogen, including names, sequences, and references. Also includes HSVd-specific primers used for grapevine/hop sequencing. |
| **Data Sheet 5**  **Data Sheet 5_Sequences for viroids and viruses** | Example sequences of the amplicons for new primer pairs | Word- Text | Lists sequences of the PCRs done for primer pair verification for viroids and viruses |
| **Table 1**  **Table 1_Primer optimization table** | Excel sheet with primer optimization details | Excel | Lists primer details along with sequences, concentration, amplicon, blast verification etc. |
| **Presentation 1**  **Presentation 6_PCR examples** | Example PCR gel pictures for viroids and viruses | Pptx | PCR gel photos showing viroid and virus detection, including the visualization of band differences across a template dilution series (1:10, 1:100, etc.) |
