# Supplementary material for: Viroid ecology in hops (Humulus lupulus L.): high prevalence in commercial systems but low presence in wild populations
Source: Front Microbiol. 2026 Jan 5;16:1652923. doi: 10.3389/fmicb.2025.1652923 (PMC12813154; doi:10.3389/fmicb.2025.1652923)

**Viroid Ecology in Hops (*Humulus* *lupulus* L): High Prevalence in Commercial Systems but Low Presence in Wild Populations**

## **Authors**

**Swati Jagani ^1^, Christina Krönauer ^2^, Ute Born ^1^, Michael Helmut Hagemann ^1^**

^1^ University of Hohenheim, Production Systems of Horticultural Crops, Emil-Wolff-Str. 25, 70599 Stuttgart, Germany

^2^ Bayerische Landesanstalt für Landwirtschaft, Institute for Crop Science and Plant Breeding, Huell 5 1/3, 85283 Wolnzach

## **Acknowledgement**

**Picture credits:** All photographs for the settlement and wild hops in this supplement were taken by Anna Vettermann (Email: anna.vettermann@web.de) and are used with permission.

**Data sheet 2: Sampling Context for Commercial, Settlement, and Wild Hop Populations**

**Commercial Hop Samples**

Commercial hop leaf samples were obtained directly from cultivated fields managed by hop farmers. Prior to field visits, farmers were contacted by email or phone to request permission for leaf sampling. To ensure spatial diversity, efforts were made to visit sites in different directions across hop-growing regions. Each sample consisted of approximately ten leaves, collected from different parts of the same plant. Samples were immediately placed in sealed plastic bags and stored on ice during transport. Information regarding the exact location and hop variety was not disclosed in this manuscript to protect the identity of the farms involved.


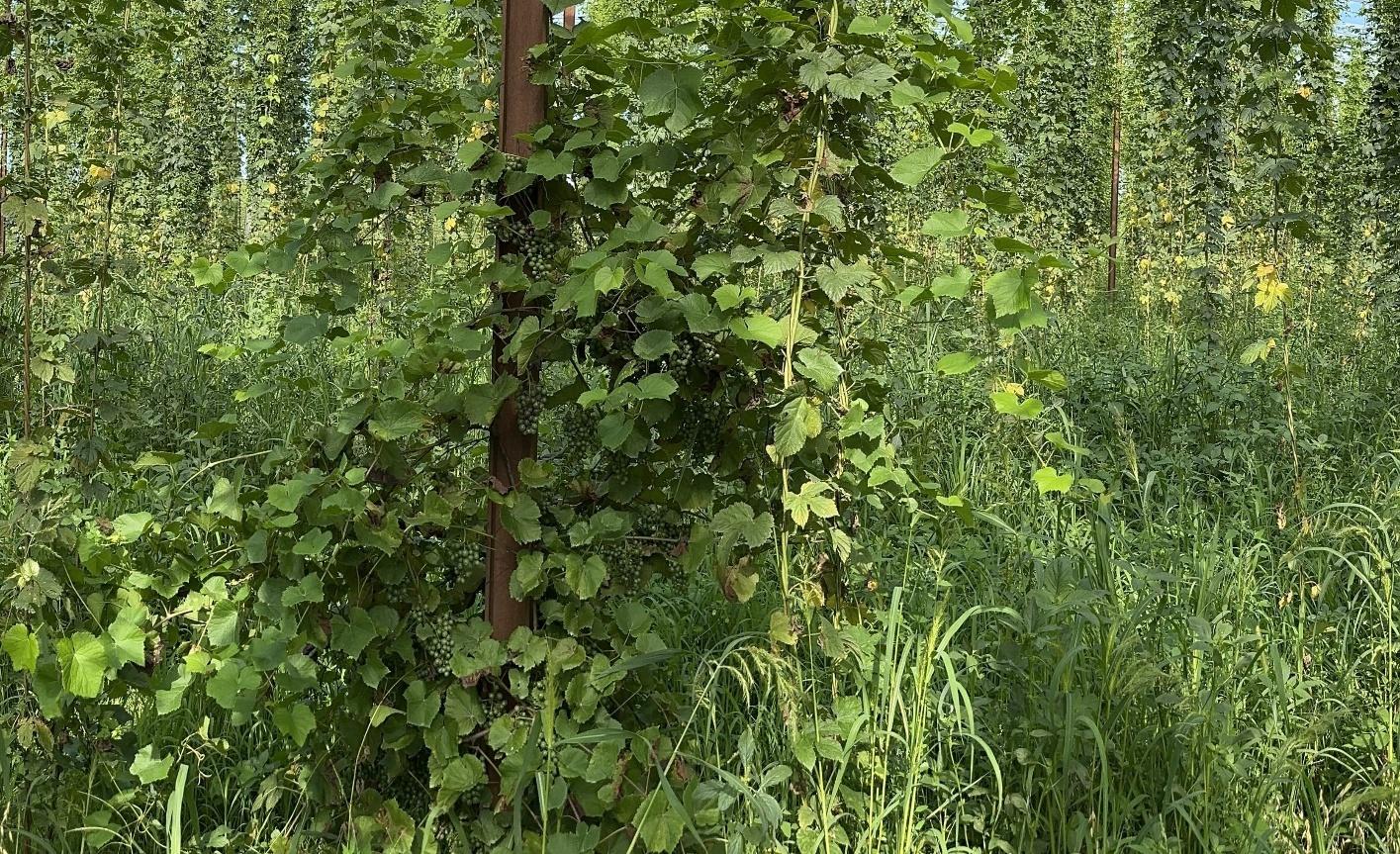


**Settlement Hop Samples**

Settlement hop plants were identified using the publicly available PlantNet app (Pl@ntNet™, CIRAD, Montpellier, France), which provides geotagged records of previously observed plant species. Each day, previously recorded hop locations were searched using the app, and field visits were made accordingly. Hops found in these areas were typically growing in semi-managed or neglected areas such as fencelines, abandoned yards, or roadside structures. Upon locating hop plants, three –seven leaves, were collected per plant. The samples were stored in sealed plastic bags on ice and brought back to the laboratory for RNA extraction and analysis.


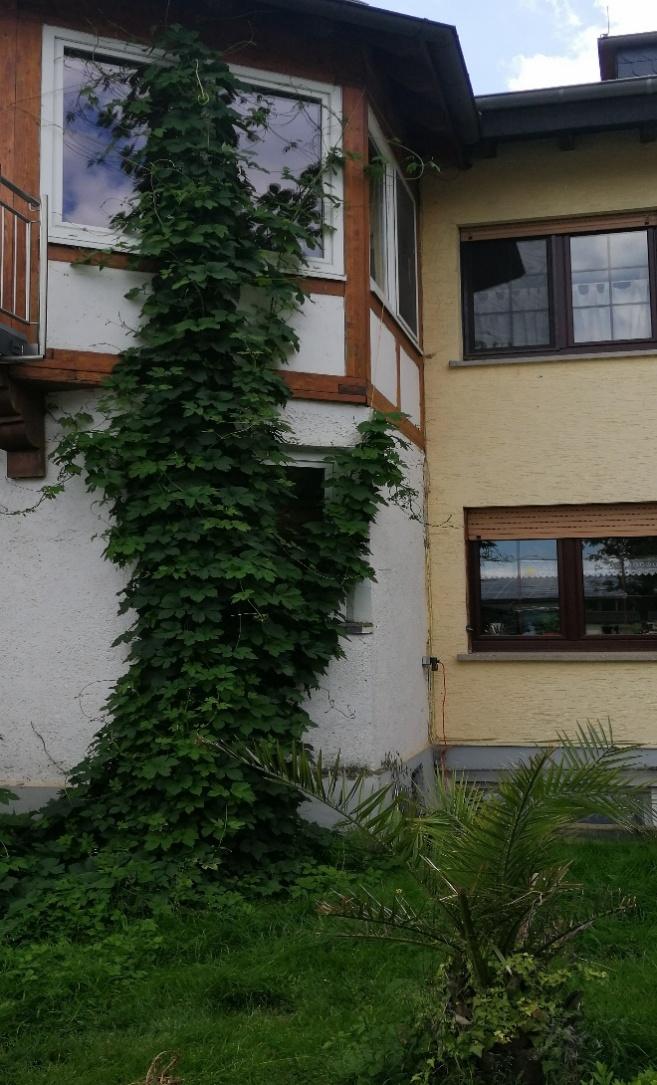

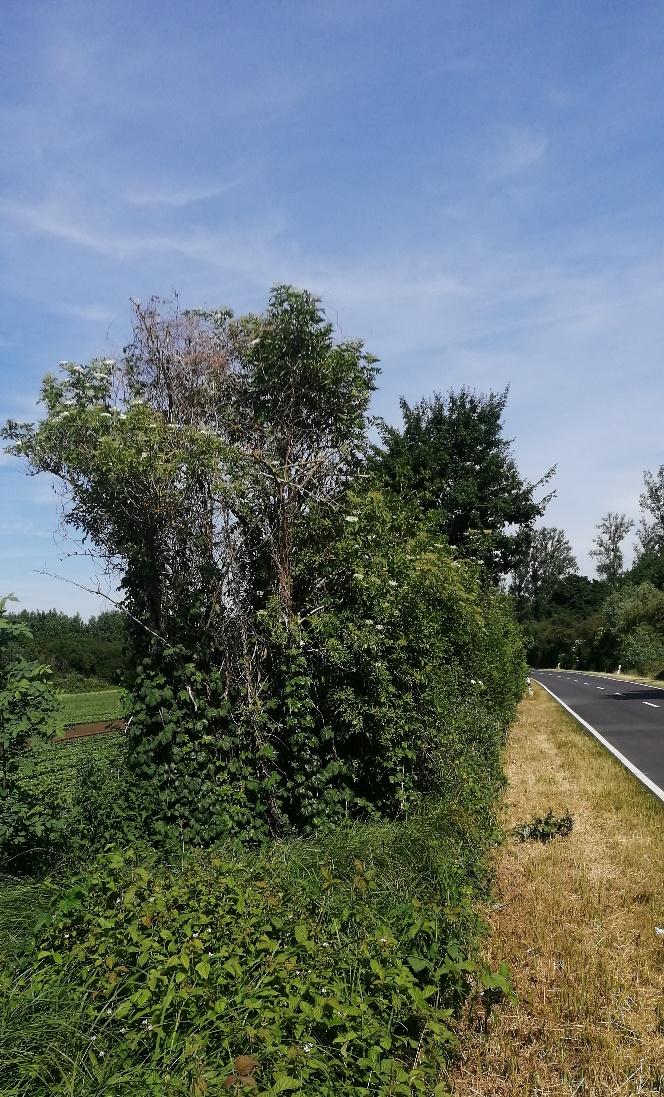


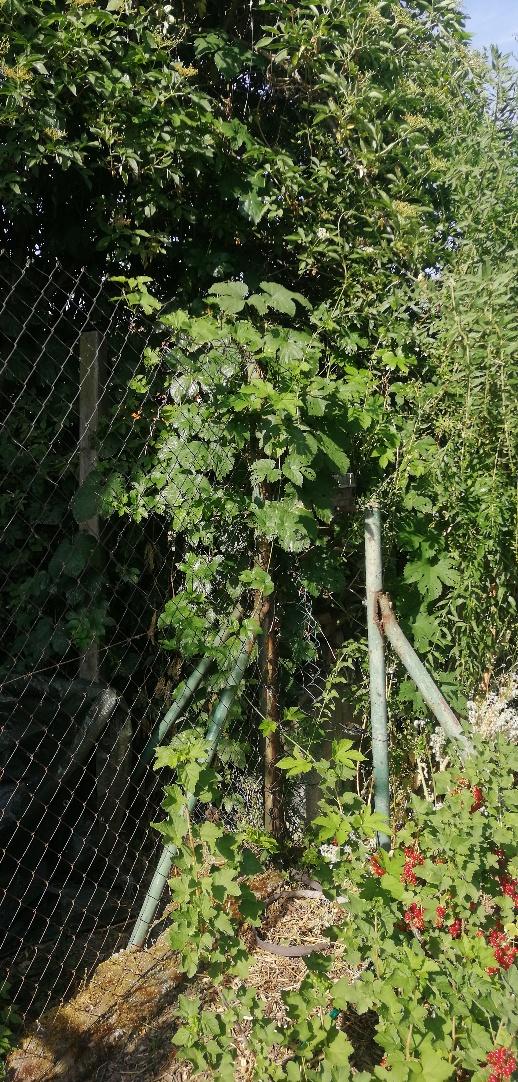

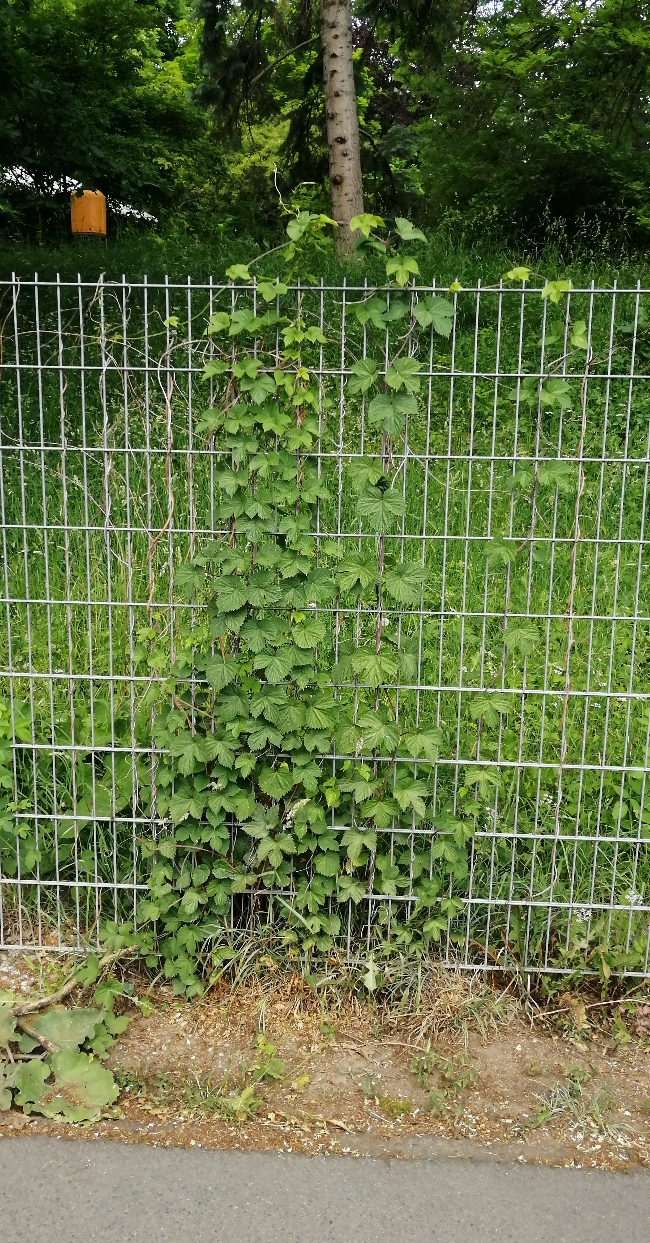


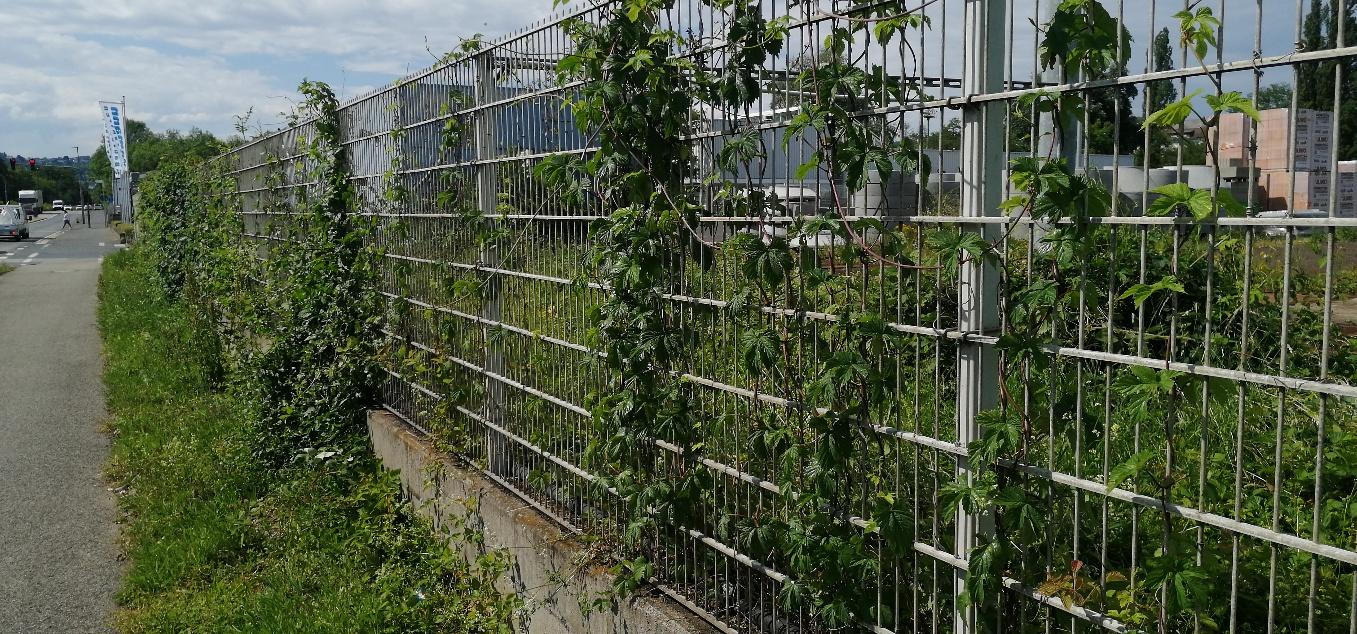


**Wild Hop Samples**

Wild hop plants were identified during the same field campaign described above. These were distinguished from settlement plants based on their naturalized location—typically found at forest edges, riverbanks, or unmanaged habitats with no visible signs of cultivation or human care. Plants showed climbing behavior on natural supports such as trees or shrubs and were often found in undisturbed vegetation zones. No disease symptoms were observed on wild plants at the time of sampling. A similar protocol was followed as for settlement samples, with leaf material collected in sterile bags and transported on ice.

All samples from commercial, settlement, and wild hops were processed individually for viroid detection and pooled for virus screening, as detailed in the Materials and Methods section of the main manuscript.


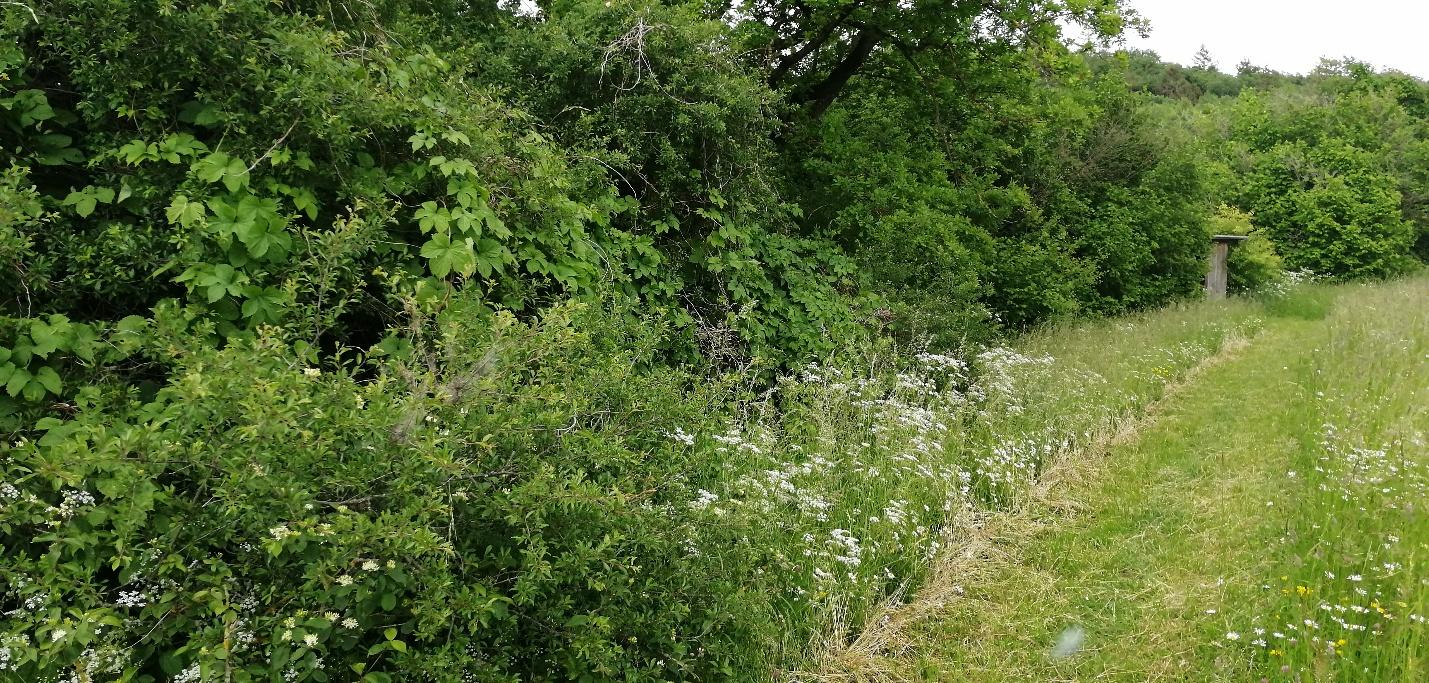


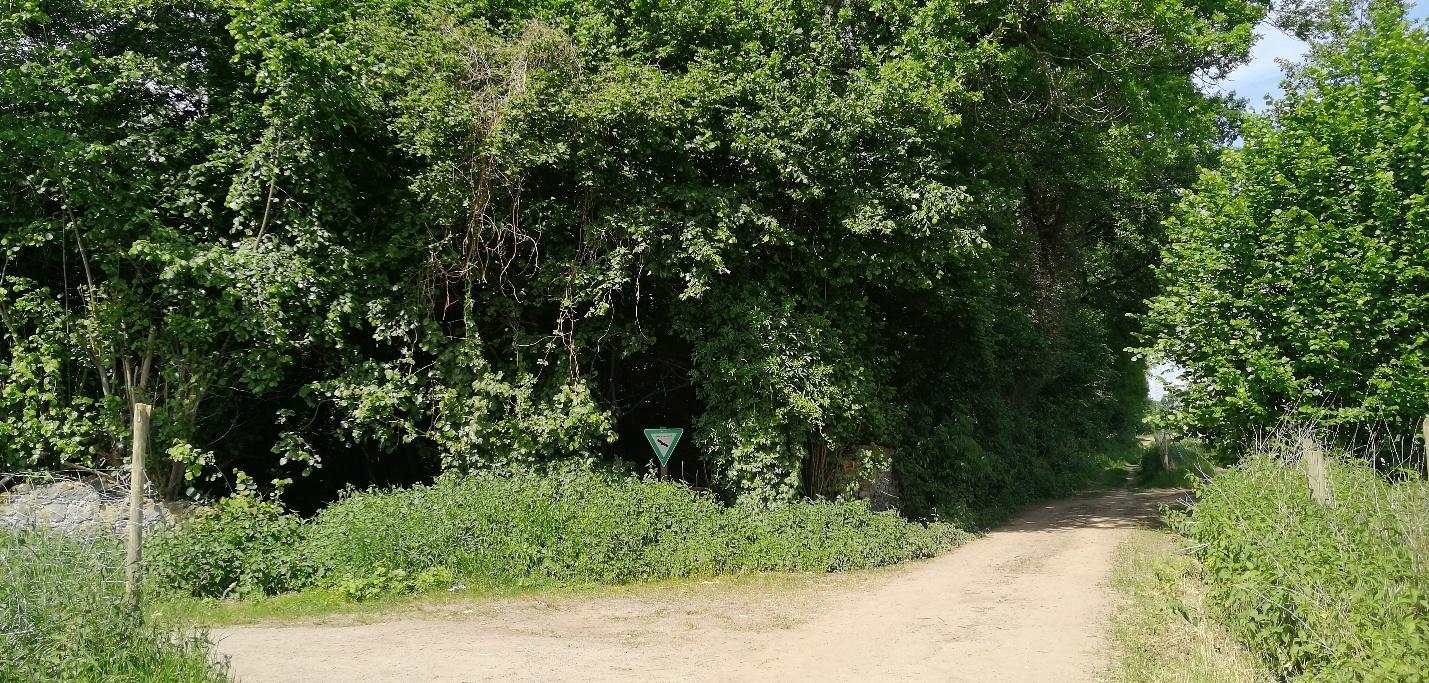


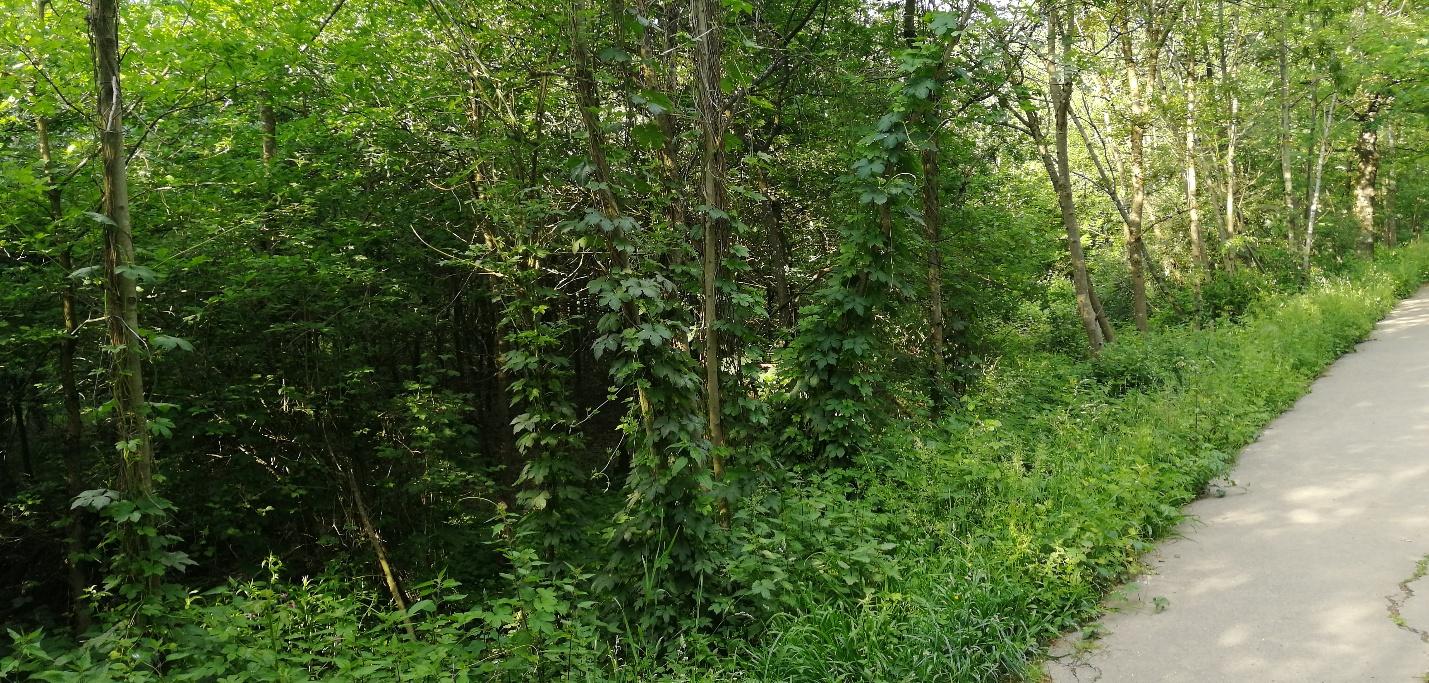


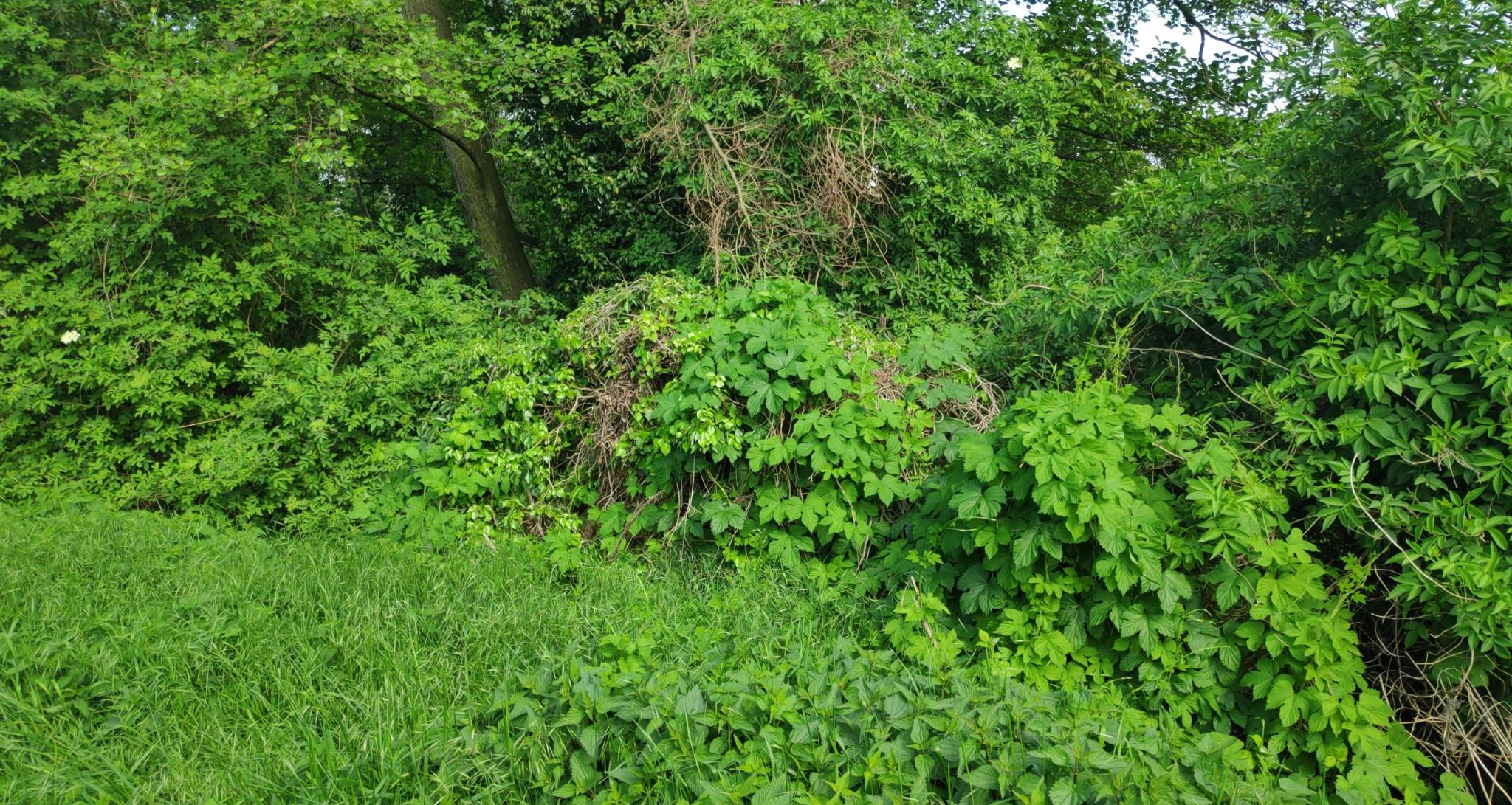


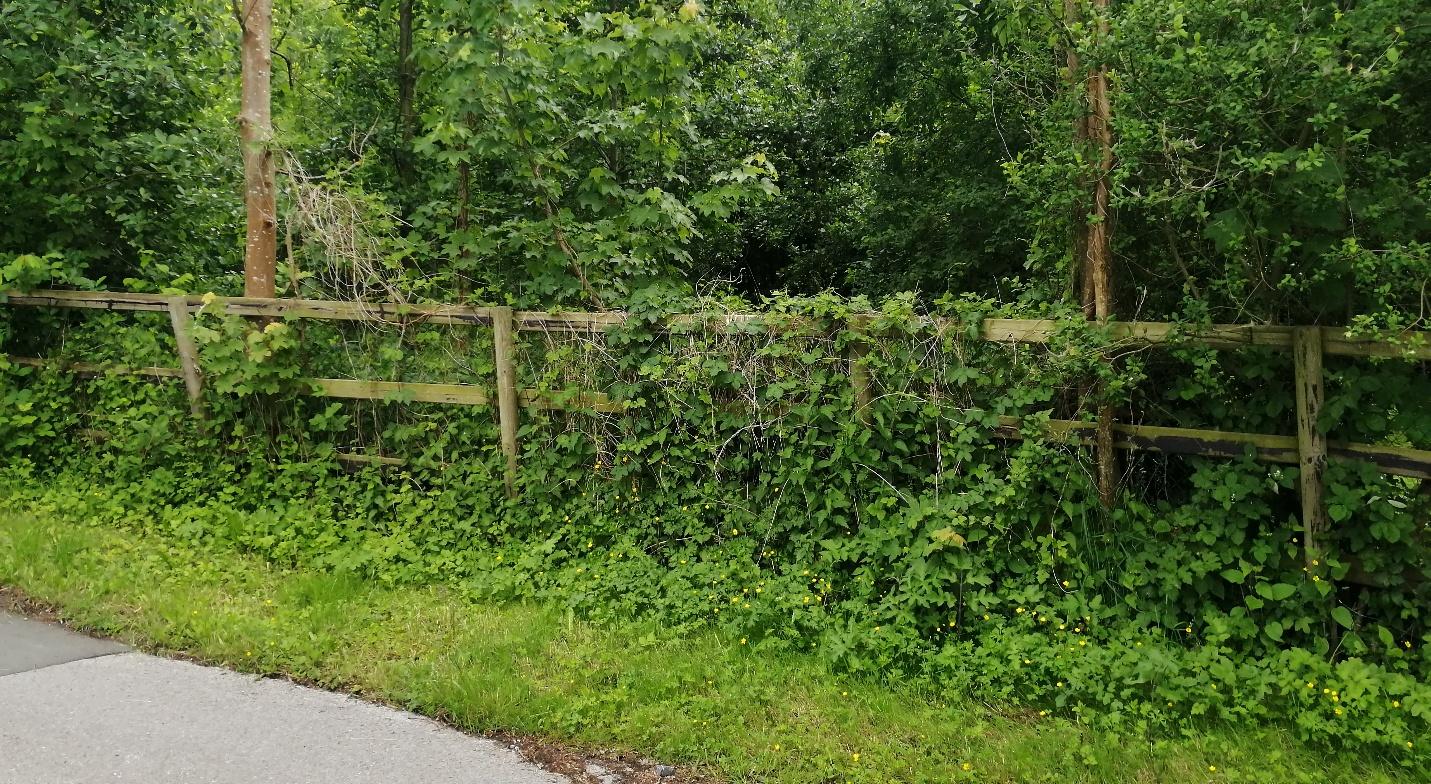


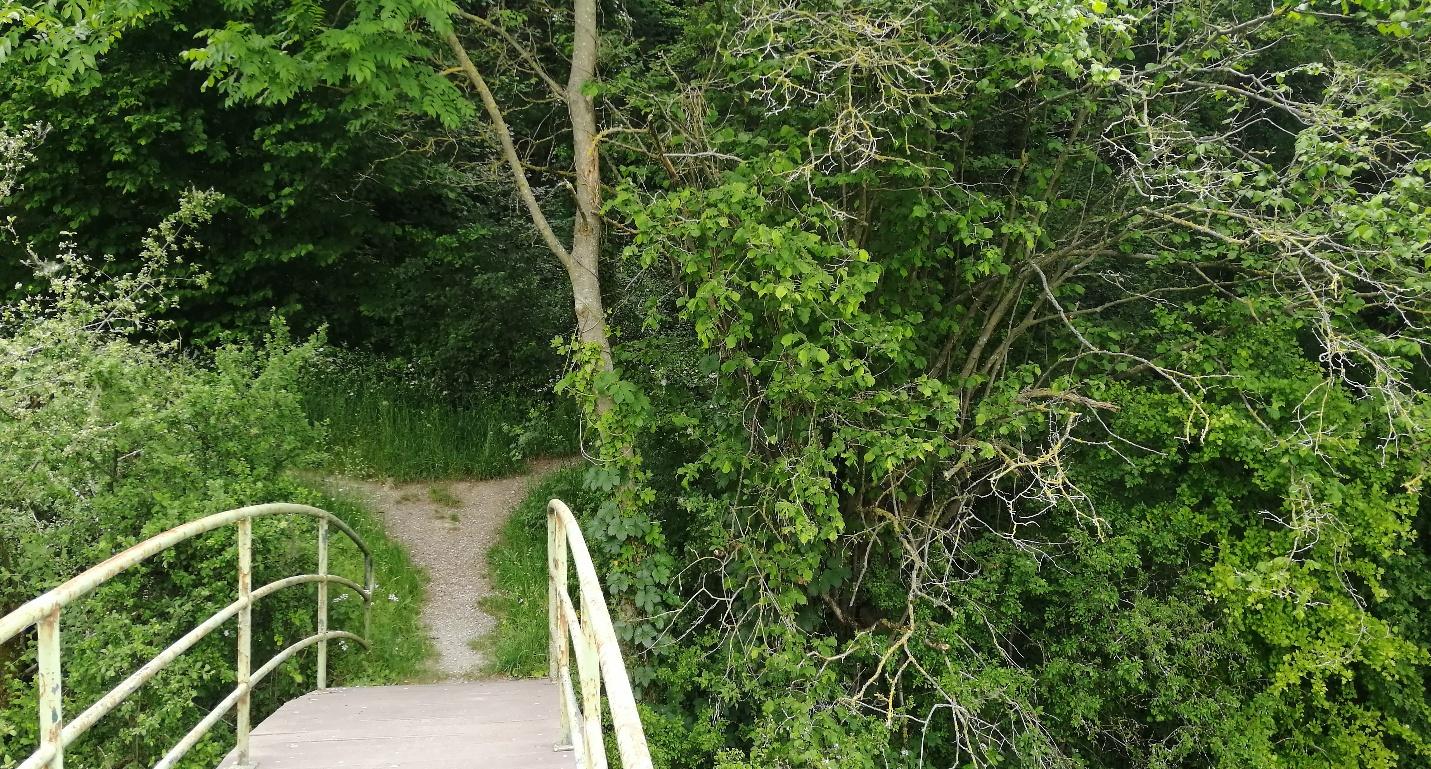

Supplement: Supplementary file 2 [file Data_Sheet_2.docx]
