# Supplementary material for: Viroid ecology in hops (Humulus lupulus L.): high prevalence in commercial systems but low presence in wild populations
Source: Front Microbiol. 2026 Jan 5;16:1652923. doi: 10.3389/fmicb.2025.1652923 (PMC12813154; doi:10.3389/fmicb.2025.1652923)
Supplement: Supplementary file 3 [file Data_Sheet_3.docx]

**Viroid Ecology in Hops (*Humulus* *lupulus* L): High Prevalence in Commercial Systems but Low Presence in Wild Populations**

## **Authors**

**Swati Jagani ^1^, Christina Krönauer ^2^, Ute Born ^1^, Michael Helmut Hagemann ^1^**

^1^ University of Hohenheim, Production Systems of Horticultural Crops, Emil-Wolff-Str. 25, 70599 Stuttgart, Germany

^2^ Bayerische Landesanstalt für Landwirtschaft, Institute for Crop Science and Plant Breeding, Huell 5 1/3, 85283 Wolnzach

# **Data sheet 3: Rationale for Pooled Virus Detection and Individual Viroid Testing**

# **Viroid Testing Strategy: Individual Sample Analysis**

To ensure maximum sensitivity in detecting viroid infections, all 418 hop leaf samples were tested individually for three viroids: citrus bark cracking viroid (CBCVd), hop latent viroid (HLVd), and hop stunt viroid (HSVd).

This approach was primarily driven by the need for high-resolution monitoring of CBCVd, a deadly and regulated viroid that causes severe stunting, bark cracking, and the eventual death of hop plants.

Part of this survey contributed to CBCVd monitoring efforts coordinated by the Bayerische Landesanstalt für Landwirtschaft (LfL), focusing on high-risk regions or areas with historical CBCVd detections. Individual testing was essential to determine whether CBCVd infections were localized or showed signs of regional spread. To maintain methodological consistency and avoid detection bias, the same individual testing strategy was applied to HLVd and HSVd.

#### **Virus Testing Strategy: Sample Pooling**

Virus testing was also conducted on the same (total of 418 samples) divided into 45 pools among three provenances (commercial, settlement and wild). Five viruses—hop latent virus (HpLV), american hop latent virus (AHpLV), hop mosaic virus (HpMV), arabis mosaic virus (ArMV), and apple mosaic virus (ApMV)—were tested using a sample pooling strategy.

The cDNA extracts of the individual samples were grouped into pools of up to 10 samples, based on population type (commercial, settlement, or wild). Pooling was applied exclusively to virus detection, following previously validated internal protocols. Presentation 1 (supplemental material) shows the validations for the sample dilutions (1:10, 1:100)

Pooling was selected for the following reasons:

- To reduce workload and costs during the large-scale screening of 418 samples.
- To capture presence/absence trends at the population level: The primary objective of virus testing was surveillance—detecting whether viruses were present within each population category (commercial, settlement, wild), rather than diagnosing individual plants.

Positive pools were not deconvoluted due to limited resources, and individual plants within positive pools were not retested. However, this was considered an acceptable trade-off, as the data still offered clear insights into virus distribution patterns between commercial, settlement, and wild populations.

Uneven pool sizes occasionally occurred due to variations in sample availability per site or context, but remained within the validated range of 5–10 samples per pool. This flexibility enabled the efficient use of available materials while maintaining detection reliability.
