# Supplementary material for: Viroid ecology in hops (Humulus lupulus L.): high prevalence in commercial systems but low presence in wild populations
Source: Front Microbiol. 2026 Jan 5;16:1652923. doi: 10.3389/fmicb.2025.1652923 (PMC12813154; doi:10.3389/fmicb.2025.1652923)
Supplement: Supplementary file 4 [file Data_Sheet_4.docx]

**Viroid Ecology in Hops (*Humulus* *lupulus* L): High Prevalence in Commercial Systems but Low Presence in Wild Populations**

## **Authors**

**Swati Jagani ^1^, Christina Krönauer ^2^, Ute Born ^1^, Michael Helmut Hagemann ^1^**

^1^ University of Hohenheim, Production Systems of Horticultural Crops, Emil-Wolff-Str. 25, 70599 Stuttgart, Germany

^2^ Bayerische Landesanstalt für Landwirtschaft, Institute for Crop Science and Plant Breeding, Huell 5 1/3, 85283 Wolnzach

**Data sheet 4: Primer table**

| **Pathogen** | **Name** | **Sequence** | **Reference** |
| --- | --- | --- | --- |
| Hop Stunt Viroid | HSVdII | GACCGGTGGCATCACCTCT | Matousek et al., 2003 |
|  | HSVdI | GCGTCTCATCGGAAGAGCC | Matousek et al., 2003 |
| Hop Latent Viroid | HLVd-M | TAGTTTCCAACTCCGGCTGG | Hataya et al., 1992 |
|  | HLVd-P | GGATACAACTCTTGAGCGCC | Hataya et al., 1992 |
| American Hop Latent Virus | AHLVcpF | ATGTCGAACGTTGAAAGG | Eastwell and Druffel, 2012 |
|  | AHLVcpR | TCAGTGCGCTTGTCGAAACTC | Eastwell and Druffel, 2012 |
| Arabis Mosaic Virus | ArMV_581_F | ACCAGTGCCTACAAGAGTGTGTCC | Komínek, 2003 |
|  | ArMV_793_R | TTGATTCCAGTTGTTAGTGACCCC | Komínek, 2003 |
| Arabis Mosaic Virus | ArMV_2378_F (new) | CTTTCGGTCCCTCATTGGCT | This study |
|  | ArMV_793_R (new) | TTGATTCCAGTTGTTAGTGACCCC | Kominek 2003 |
| Hop Latent Virus | HpLV_MH_F2 | TGCAGGGGGAAGCAAAGAAT | This study |
|  | HpLV_MH_R | TGCAACAGCAAAGCGACAC | This study |
| Hop mosaic virus | HpMV_MH_F | CAGGAAAGCTTGCCCCTGAG | This study |
|  | HpMV_MH_R | CACGGATAATCTCTGGGCCA | This study |
| Apple mosaic virus | ApMV_Fb_5 | ATGGTCTGTAAGTTCTGTGGT | This study |
|  | ApMV_1994_ | CTAATCGAGTCTCGGGGTCC | This study |
| nad | nad5_Menzel_sense (=nad5_F) | GATGCTTCTTGGGGCTTCTTGTT | Menzel et al., 2002 |
|  | nad5_Menzel_antisense (=nad5_R) | CTCCAGTCACCAACATTGGCATAA | Menzel et al., 2002 |

**Primer list for HSVd sequencing for hop and grapevine**

| HSVd | HSVdSano9F (original: HSV-9) | CGCGGTGCTCTGGAGTAGA | Nakahara K., et al. 1998 |
| --- | --- | --- | --- |
|  | HSVdSano9R  (original : HSV 8-M) | CGCCTCTCGCTGGATTCTG | Nakahara K., et al. 1998 |
|  | HSVd_Eich_F | GGGCAACTCTTCTCAGAATCC | Eichmeier et al. 2016 |
|  | HSVd_Eich_R | GTTGGAAGACGAACCGAGAG | Eichmeier et al. 2016 |
|  | HSVdII | GACCGGTGGCATCACCTCT | Matousek et al. 2003 |
|  | HpSVd3-160 | GACGATCGATGGTGTTTCGAAG | Ziegler et al. 2014 |
|  | HSVdF2_JK (or JK_F) (extra primer pair not used for full length) | GACTTACCTGAGAAAGGAGCCC | Hagemann et al., 2023 |
|  | HSVdR2_JK (or JK_R) (extra primer pair not used for full length) | ACAAAAAGCAGGTTGGAAGACG | Hagemann et al., 2023 |

**References**

Matousek, J., Orctová, L., Patzak, J., Svoboda, P., & Ludvíková, I. (2003). Molecular sampling of hop stunt viroid (HSVd) from grapevines in hop production areas in the Czech Republic and hop protection. Plant Soil and Environment, 49(4), 168-175.<https://doi.org/10.17221/4109-pse>

Ziegler, A., Kawka, M., Przybys, M., Doroszewska, T., Skomra, U., Kastirr, U., et al. (2014). Detection and molecular analysis of hop latent virus and hop latent viroid in hop samples from Poland. Journal für kulturpflanzen <https://doi.org/10.5073/JfK.2014.07.04>

Eichmeier, A., Kominkova, M., Pecenka, J., & Kominek, P. (2019). High-throughput small RNA sequencing for evaluation of grapevine sanitation efficacy. Journal of virological methods, 267, 66-70.<https://doi.org/10.1016/j.jviromet.2019.03.003>

Nakahara, K., Hataya, T., & Uyeda, I. (1998). Inosine 5′-triphosphate can dramatically increase the yield of NASBA products targeting GC-rich and intramolecular base-paired viroid RNA. Nucleic acids research, 26(7), 1854-1855. <https://doi.org/10.1093/nar/26.7.1854>

Eastwell, K. C., & Druffel, K. L. (2012). Complete genome organization of American hop latent virus and its relationship to carlaviruses. Archives of Virology, 157(7), 1403–1406. <https://doi.org/10.1007/s00705-012-1312-0>

Kominek, P., Svoboda, P., & Abou Ghanem-Sabanadzovic, N. (2003). Improved detection of Arabis mosaic virus in grapevine and hop plants. Acta virologica, 47(3), 199-200.

Menzel, W., Jelkmann, W., & Maiss, E. (2002). Detection of four apple viruses by multiplex RT-PCR assays with coamplification of plant mRNA as internal control. Journal of Virological Methods, 99(1-2), 81-92.<https://doi.org/10.1016/S0166-0934(01)00381-0>

Hagemann, M. H., Treiber, C., Born, U., Schrader, G., Stampfl, J., Jakše, J., & Radišek, S. (2023). Risk potential of international fruit trade for viroid spreading-case study on hop viroids in Europe. Journal of Plant Pathology, 105(4), 1335-1346. <https://doi.org/10.1007/s42161-023-01449-3>

Hataya, T., Hikage, K., Suda, N., Nagata, T., LI, S., Itoga, Y., et al., (1992). Detection of hop latent viroid (HLVd) using reverse transcription and polymerase chain reaction (RT-PCR). Japanese Journal of Phytopathology, 58(5), 677-684. <https://doi.org/10.3186/jjphytopath.58.677>
