# Supplementary material for: Viroid ecology in hops (Humulus lupulus L.): high prevalence in commercial systems but low presence in wild populations
Source: Front Microbiol. 2026 Jan 5;16:1652923. doi: 10.3389/fmicb.2025.1652923 (PMC12813154; doi:10.3389/fmicb.2025.1652923)
Supplement: Supplementary file 5 [file Data_Sheet_5.docx]

**Viroid Ecology in Hops (*Humulus* *lupulus* L): High Prevalence in Commercial Systems but Low Presence in Wild Populations**

## **Authors**

**Swati Jagani ^1^, Christina Krönauer ^2^, Ute Born ^1^, Michael Helmut Hagemann ^1^**

^1^ University of Hohenheim, Production Systems of Horticultural Crops, Emil-Wolff-Str. 25, 70599 Stuttgart, Germany

^2^ Bayerische Landesanstalt für Landwirtschaft, Institute for Crop Science and Plant Breeding, Huell 5 1/3, 85283 Wolnzach

# **Data sheet 5: Amplicon Sequences of Hop Viruses and Viroids**

This file contains the examples of nucleotide sequences of the amplicons generated using the primer sets described in the Methods section and (Data sheet 4). Sequences are provided in FASTA format, with each entry labeled by pathogen name and amplicon identifier.

**Viroids**

**˃Hop_Stunt_Viroid_Amplicon, Partial |Primers: HSVd_II and HpSVd3_160_**

ggacgatcgaaatgtgtttcgaaggcagagcctctactccagagcaccgcggccctctctccacgcctctcgctggattctgagaagagttgccccggggctcctttctcaggtaagtacctccctgccttgttttttctttgcttggccttctgcggcaactcgagaattccccagaggggctcaagagaggatccgcggccagaggctcagatagacaaaaagcaggttgggacgaaccgagaggtgagcccaccgg

**˃Hop_Stunt_Viroid_Amplicon, Partial | Primers: HSVdSano9F and HSVdSano9R**

ctgagaagagttgccccggggctcctttctcaggtaagtacctccctgccttgttttttctttgcttggccttctgcggcaactcgagaattccccagaggggctcaagagaggatccgcggccagaggctcagatagacaaaaagcaggttgggacgaaccgagaggtgatgccaccggtcgcgtctcatcggaagagccagaagaaggtaaagaagaagggacgatcgatggtgtttcgaaggcagagcctcta

**˃Hop_Stunt_Viroid_Amplicon, Partial | Primers: HSVd_Eich_F and HSVd_Eich_R**

gtgatgccaccggtcgcgtctcatcggaagagccagaagaaggtaaagaagaagggacgatcgatggtgtttcgaaggcagagcctctactccagagcaccgcggccctctctccacgcctctcgctggattctgagaagagttgcc

**˃Hop_Latent_Viroid_Amplicon, Partial | Primers: HLVd-M and HLVd-P**

gtgtgaagaaggagccgttccacgcaggacgcgaacaagaagaagccgaagcaacttcaggtcgccgcgcacgaactggcgctcgatctccgcctcgctcgagtaggtttccccggggatccctcttcgagcccttgccaccatacaggtaagtcacgtagtgtattccccagaggggcactttttatgtgaacttctgcaggtaaagctc

**Viruses**

**˃Hop_Mosaic_Virus_Amplicon, Partial | Primers: HpMV_MH_F and HpMV_MH_R**

ggccactgcggccaccggtaacctctacatccatattcccaaaatgctgattcctgttggctctccggagggcgatatccttgtgcgtgctgtgcgctaacttctccctcggcgttggacgtctcactatgccgtcaagtggttgcacagcagcggcattctcaacataatcgaagcagtcgaaagctgcgaaacggtcctcaaactgaaatcccatcgccgcccaatcagaaggtggctggttgtgcaccaacatgtaagaccacgtcacgggggcatacaagcgacagaccctgcggagtgtctcagcatctttcttcatgatagctagcactgagtctgctgcaatggctccgccaggccactcaaatgaaccccttgggtctaaatacacagagctgctcgcgtccttacagtagatcacggcctgaataataaccctttgtatgtgctcagttggaacacctagcccttctaagttcgtataaatcttcattatatcctctgcagtggccatgttgttggaaacaggtttcggtggtatacgcagcagtgagtcaagagacggtttacagtaggggttggcagggtcagagcgcatatcagctgtaagtaccaacgccggccgacctgtttctaaactggaattcgtaatttgcacggcactgcgcctcctacgcataaattctctcagcttctctaacctcgcttccaacatttcttcccgcacagcttccagatctttgagcccagcggcatagtcatccacgctcacggttgaggactcaccggctcccactttctctttaagtttggtctcttccccagcgtttttcggcttttgggtctcagg

**˃Hop_Latent_Virus_Amplicon, Partial |Primers: HpLV_MH_F2 and HpLV_MH_R**

acacgcaggtaaaacatacttaattattagacttagagtaatcacgttgaagctcgggcccattctttcccccggttatttccacatctagattcccgaaaagctgattcctattcgcacggcgcagcgctagatctttatgagtcttatttgcaatcttctctcttgctgtggggactctgatcagcccttccagtggttgcacagccgcagcattttcaacgtagtccaagcaatcaaaagctgcaaagcgatcttcacgctgaaagcccatttcggaccagtcagaagggggattgttatgtagcagcatgtagttccacgtgagtggcgcatacaacctgcaaacgcgcctcaaggtctcggcatccttctttattatcgcaagcactgaatcggccgagatagccccgccacgccactcgaaagagccccgggggtccacatagggtgaactgctggagtccttgcaatagaacaccgcttgcaagatcacgctttgcacgtgttcagtgggcacacccaacccctctagatcaacgcagatcttcatcatatcctccgaggtggccatgttgtttgacacgaccctaggctttatcatcaacaaagcatccaacgagggtttgttgtacgggttggtaggatcaggcagcatgtcgggtgtaggcttgagtgcgggccggcctgtttctagccctgcgttagtcacgcgaatagctgcccttctacgagccaggaactccctcaatttgctcaacctcaactccaccatctcctcttcatcatcatcagaccctagcccaggatcaatgtacttcttaagcaactcatctttcctctttgaggactcagccttttctttattct

**˃Apple_Mosaic_Virus_Amplicon, Partial | Primers: ApMV_Fb_5 and ApMV_1994_**

ttggtccatcccccctaaaacgcatatcctctcgatatgcttcagttccctttcggaactttgagcttcacgctcctacagttctaacaaatcctcatcgataagtagaacattcatcggtatttgcaccggtggtaactcactcgttatcacatacaaatccctcatgaagactttcgcaccagcggcgaaattcgtcttaaactccaaaaccaatctaaacttcctcacgaagtcatcaaaggttgtgtttggaggggcttcccactgccaacctctcggttggtccttcttgaagccttttcggtccatcggattcggacctttcggattactctcatcgtaatcctctactagaccaatccacccattagaggatagactgtttactcggattatgaaggtatacaccttcaaatccttctctagcagatcacggaagacgtcggcaaagtcaatattcacgaatttgcctgcaaccgtagcagtcacttctcgactgctcaaagccctgtgaccctttggaatctttggttccacattcgggcctctaacctcccaagctgtcctcctcacggacggacctcccttcacgggattcttacccttgttcgggttcgcttgggcgcgaataacagcgcgcttcggaggagcgggtctattcgttccgtgacaccacttgcaattaatgcaagctccagggtgagtgtg

**˃American_Hop_Latent_Virus_Amplicon, Partial | Primers: AHLVcpF and AHLVcpR**

aactccctcttaattgcaggacctcgcattcccccagtaatctcggtgtcgtaactcccgaaagcctcatcccgtgcattcctatccaaagccaggcgttggtgcgtcttgtgagctatgcactcagcactagttgggtgtcttatgagtccctcgactggttgaatcgcagctgggttgaggatataatcgaaggtatcgaaagcggcgaagcgctcttcgtacctgaaacccatcgcttgccagtcagacggaggcgtgttgtgcagcaacatgtagttccagacgatcggggcgtacaagcgagagaccttcctggccgtgctcctcttgttgaatatggcatgtagggaatcgcggggaatagcaccagtcccaaaagatatagacccgttgggatccatgtacctcgagctgctcgtgtttgcgtagtagaggaccatctgtatgatcacccgcgtgacgtgctctgtcgggaccccagcaccttcaatgtcagagaagatcagagcaatgtgctccgaagtggccatattggttgaattcgcttctggcggtaaggcaatcaagtgctcaagtgaaagcatagcgtatggattggcgagatctggtgtcatctccgctagcggcctcagtctgggtctgcccttctcataactcatgttcttgacaatgacactcatgcgctcacttctgactgaatctaaaagctcatccagcctcttaacttcttctgcagccagaatctgagcaggtgatggcgtcaccacgtgtcttggggcatttggcaggtcattccccggggttttggctccactcgaagctggagcaggcgggacgacgacctggtggtccttgtcgcgcggttctcggcttggggttggcgggacctcggtttc

**˃Arabis_Mosaic_Virus_Amplicon, Partial | Primers: ArMV_2378_F_ and ArMV_793_R_**

tttgattcccagtgttagtgaccccgttccattcactaacaactcttaggactatgccataagtacaagggctacatatatgcactttgcccttgaccacaccaccaataccaagataataactcaaaagtgtattgttataattatacactttatgagttccatcaacaagagtttgacccaacagaagggatacactcttgtaggcactggtagtacttaggtcatattccagtggtgggatagtcagaaacttgaaagacccattgaaagcatcagcgctataaatgaaattgggtcttccaaggaaagtttttgctgcctcaaattcagcatatagctcaactacaaactcccaatctgcagctagctctttgttgtttccagtgaggcatgtgaaatggagtttggggctctcaaaagtagtggcgttaaaccacatagcatgtccacagagctccccataatcaatatcacatgaggttgtcccatttttatgatgcaacagccaatgaggaacccc
