# Supplementary material for: Viroid ecology in hops (Humulus lupulus L.): high prevalence in commercial systems but low presence in wild populations
Source: Front Microbiol. 2026 Jan 5;16:1652923. doi: 10.3389/fmicb.2025.1652923 (PMC12813154; doi:10.3389/fmicb.2025.1652923)
Supplement: Supplementary file 6 [file Presentation_1.PPTX]

## Slide 1
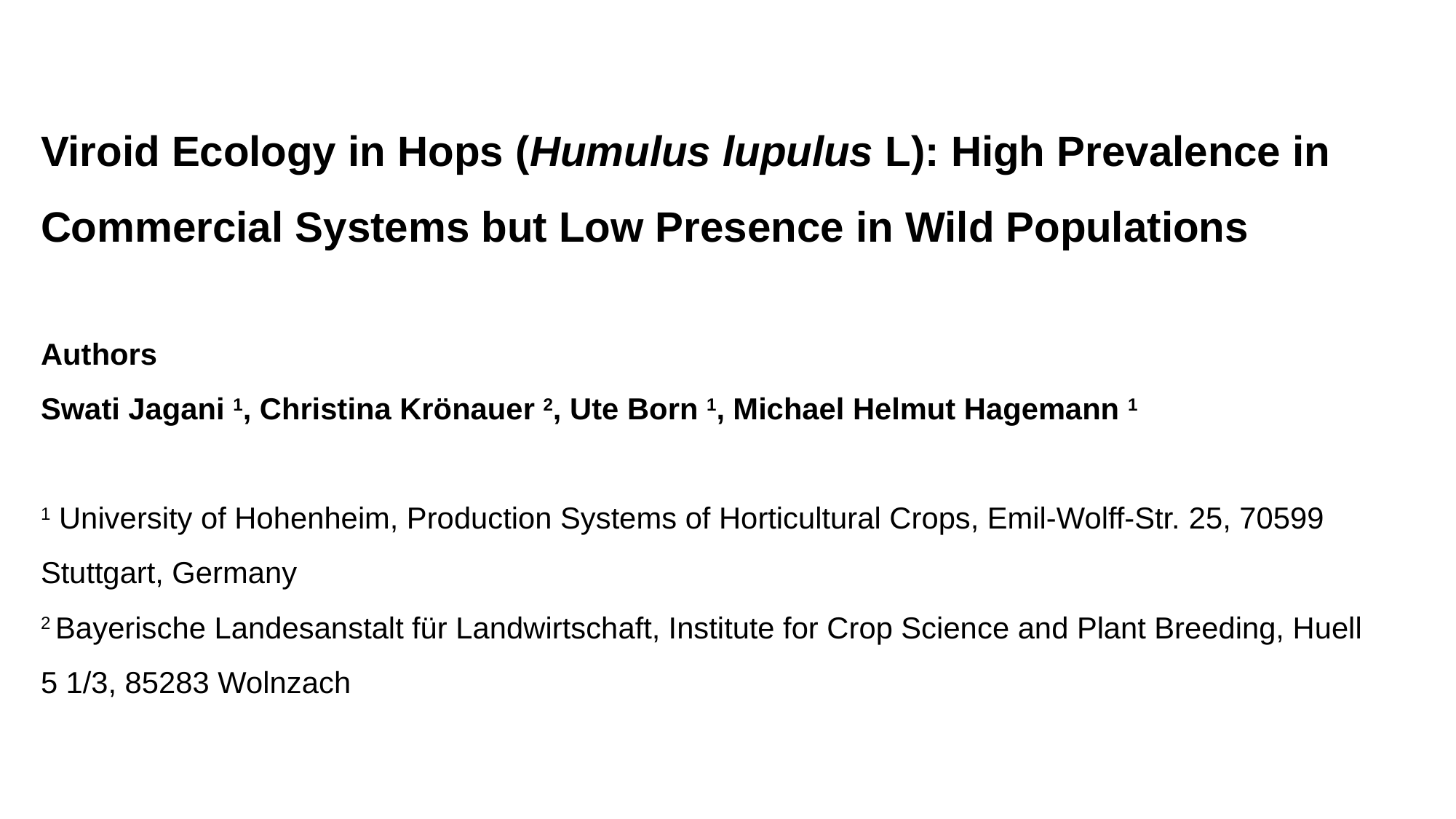

# Viroid Ecology in Hops (Humulus lupulus L): High Prevalence in Commercial Systems but Low Presence in Wild Populations Authors Swati Jagani 1, Christina Krönauer 2, Ute Born 1, Michael Helmut Hagemann 1 1 University of Hohenheim, Production Systems of Horticultural Crops, Emil-Wolff-Str. 25, 70599 Stuttgart, Germany 2 Bayerische Landesanstalt für Landwirtschaft, Institute for Crop Science and Plant Breeding, Huell 5 1/3, 85283 Wolnzach

## Slide 2
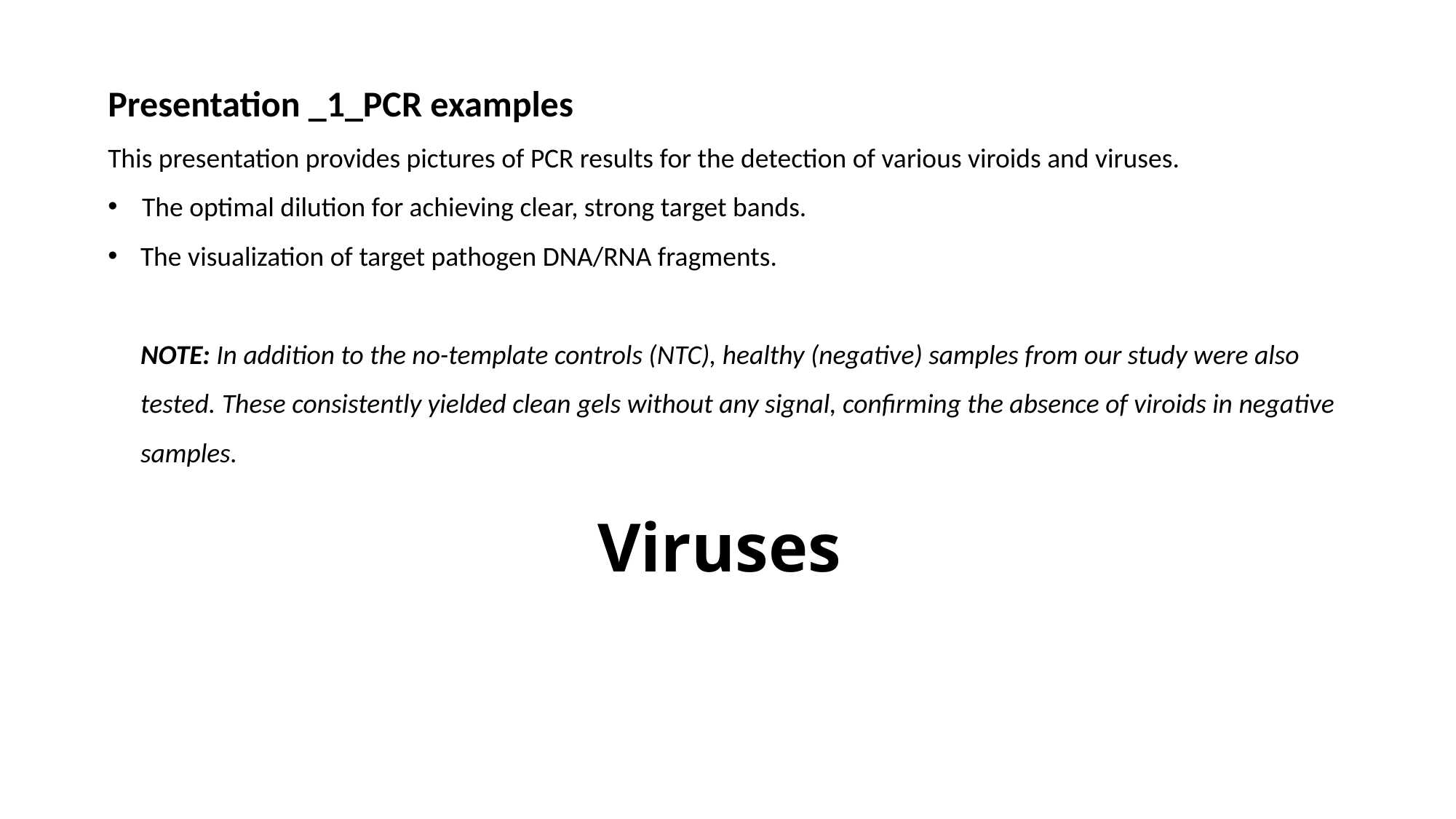

Presentation _1_PCR examples
This presentation provides pictures of PCR results for the detection of various viroids and viruses.
The optimal dilution for achieving clear, strong target bands.
The visualization of target pathogen DNA/RNA fragments.
NOTE: In addition to the no-template controls (NTC), healthy (negative) samples from our study were also tested. These consistently yielded clean gels without any signal, confirming the absence of viroids in negative samples.
# Viruses

## Slide 3
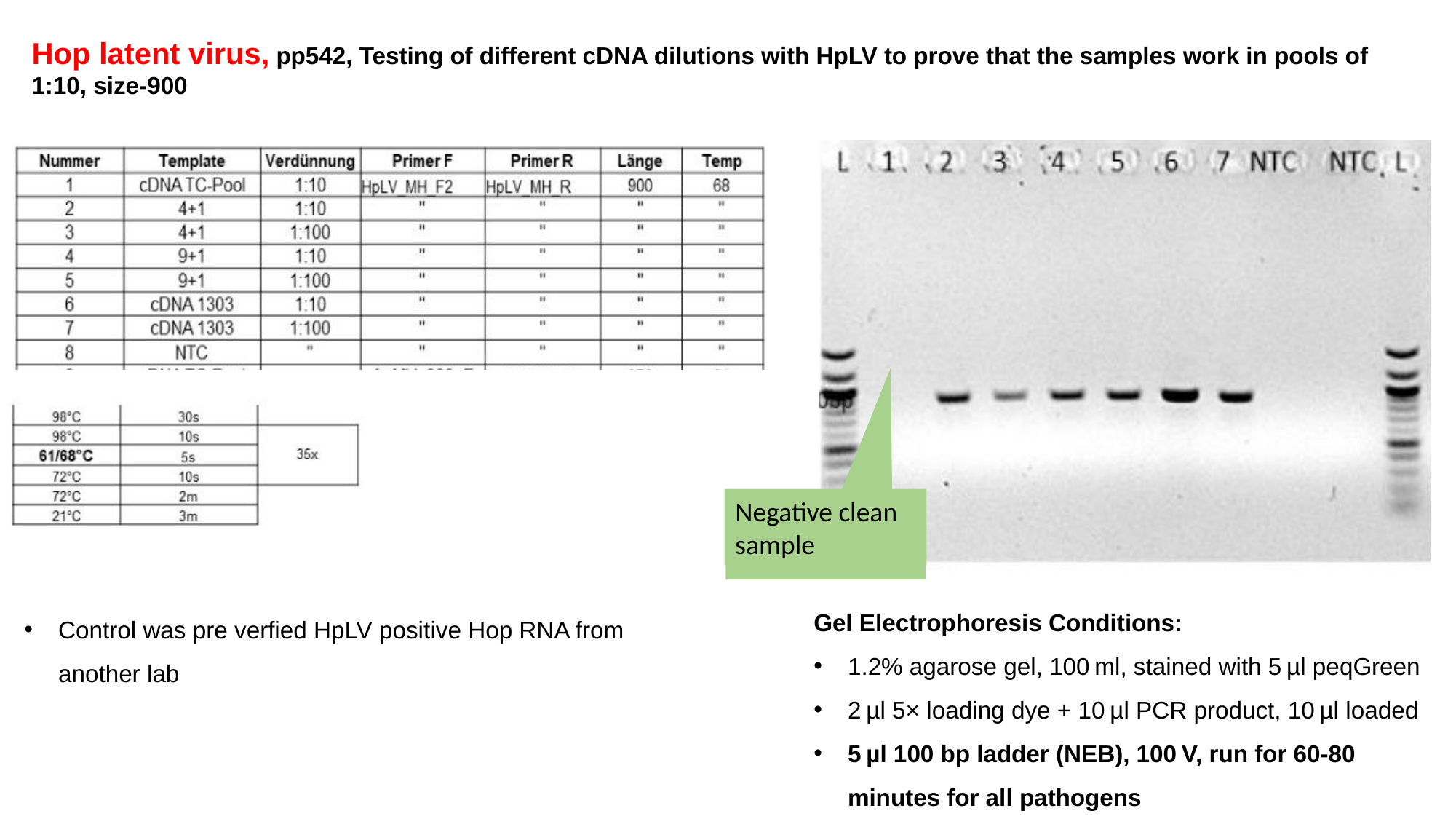

Hop latent virus, pp542, Testing of different cDNA dilutions with HpLV to prove that the samples work in pools of 1:10, size-900
Negative clean sample
Gel Electrophoresis Conditions:
1.2% agarose gel, 100 ml, stained with 5 µl peqGreen
2 µl 5× loading dye + 10 µl PCR product, 10 µl loaded
5 µl 100 bp ladder (NEB), 100 V, run for 60-80 minutes for all pathogens
Control was pre verfied HpLV positive Hop RNA from another lab

## Slide 4
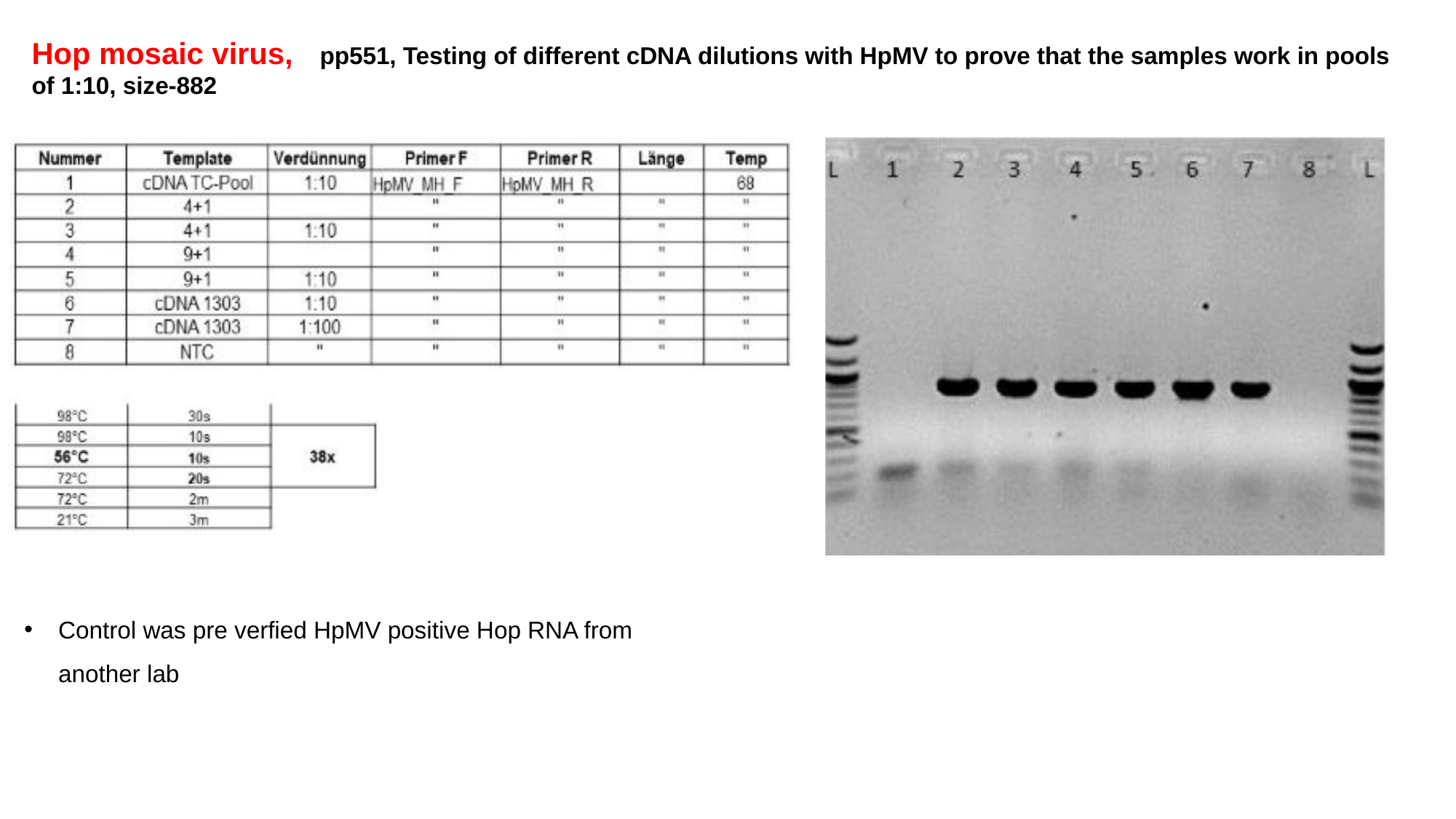

Hop mosaic virus,    pp551, Testing of different cDNA dilutions with HpMV to prove that the samples work in pools of 1:10, size-882
Control was pre verfied HpMV positive Hop RNA from another lab

## Slide 5
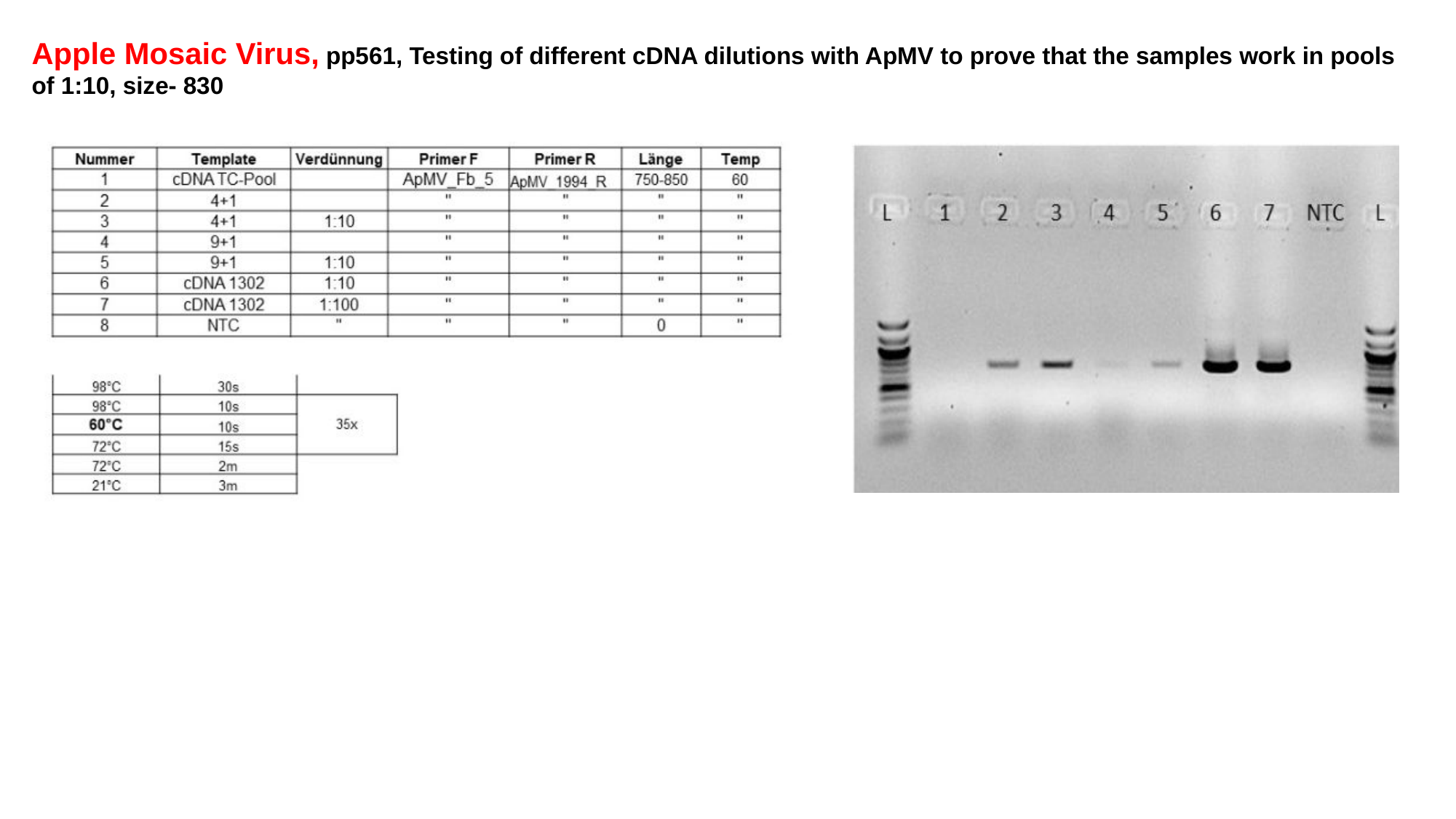

Apple Mosaic Virus, pp561, Testing of different cDNA dilutions with ApMV to prove that the samples work in pools of 1:10, size- 830

## Slide 6
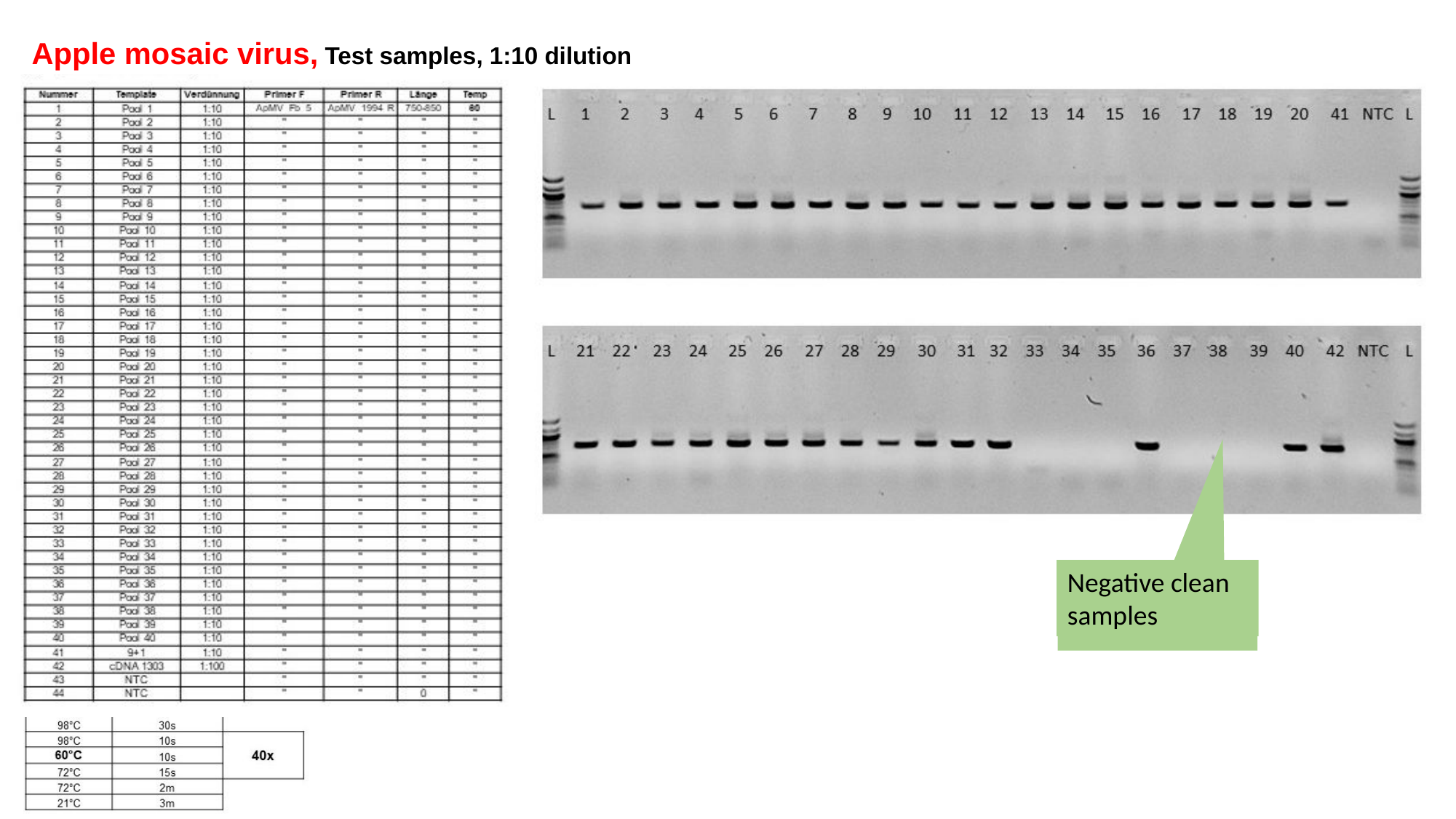

Apple mosaic virus, Test samples, 1:10 dilution
Negative clean samples

## Slide 7
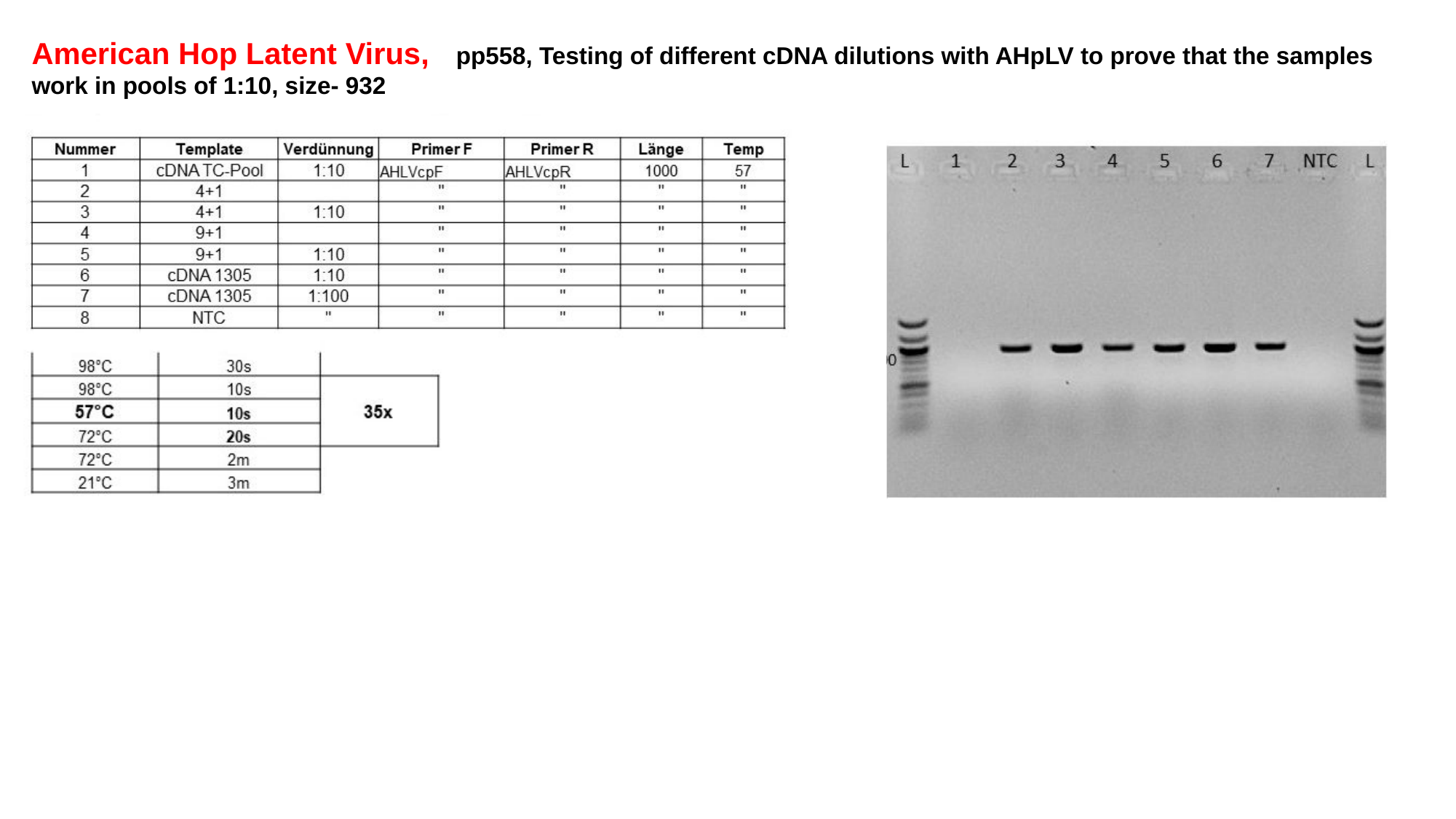

American Hop Latent Virus,    pp558, Testing of different cDNA dilutions with AHpLV to prove that the samples work in pools of 1:10, size- 932

## Slide 8
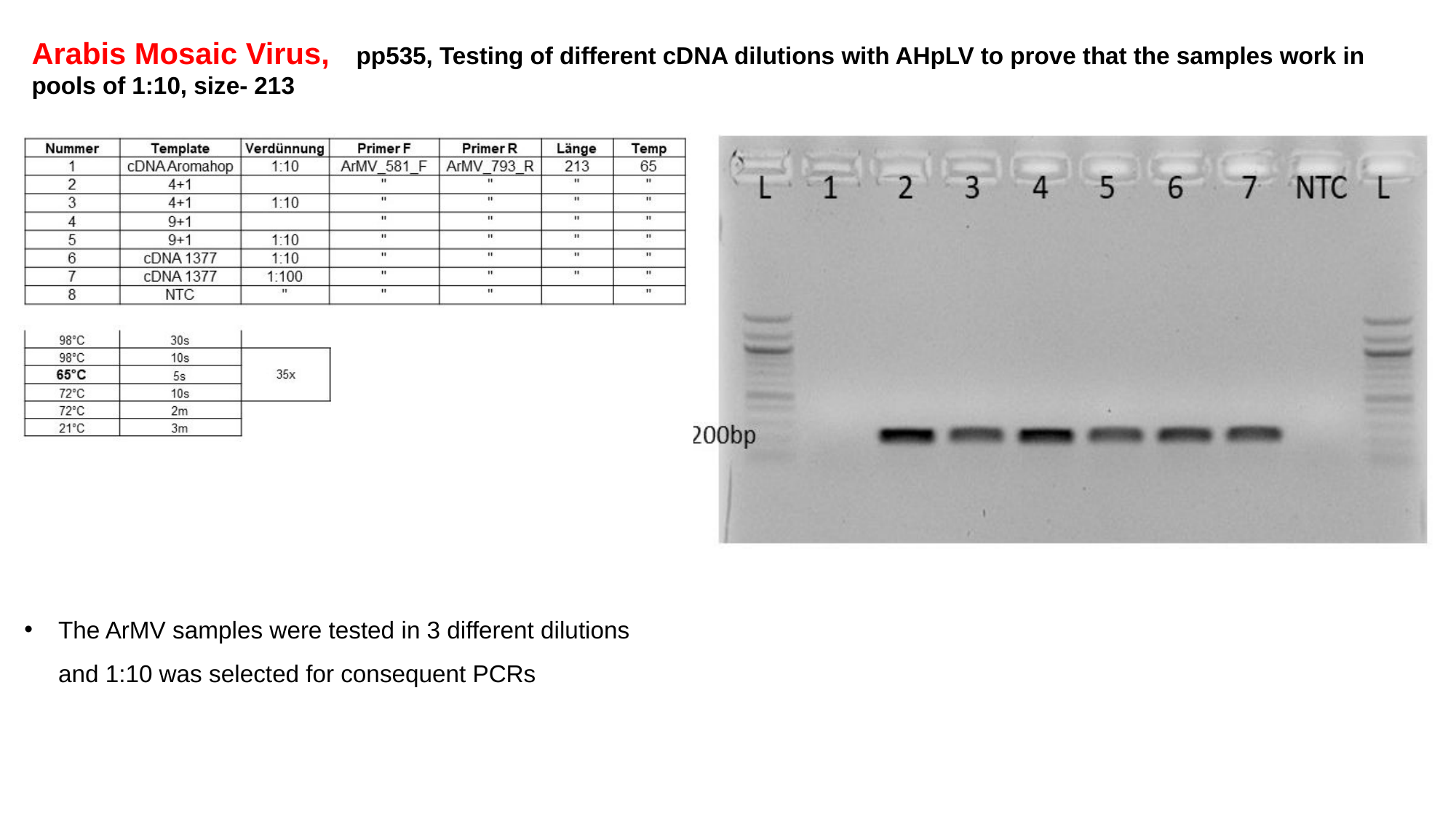

Arabis Mosaic Virus,    pp535, Testing of different cDNA dilutions with AHpLV to prove that the samples work in pools of 1:10, size- 213
The ArMV samples were tested in 3 different dilutions and 1:10 was selected for consequent PCRs

## Slide 9
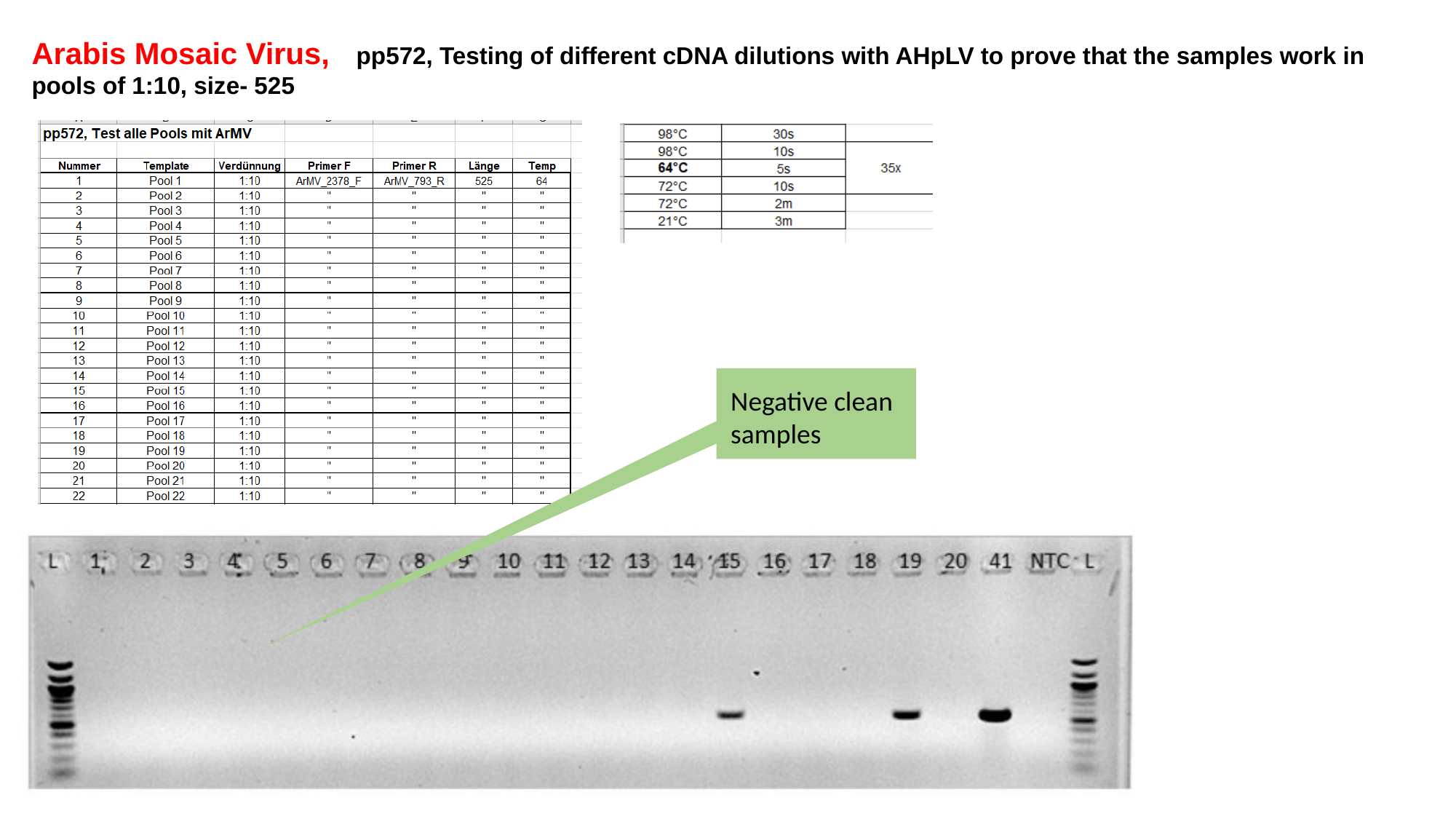

Arabis Mosaic Virus,    pp572, Testing of different cDNA dilutions with AHpLV to prove that the samples work in pools of 1:10, size- 525
Negative clean samples

## Slide 10
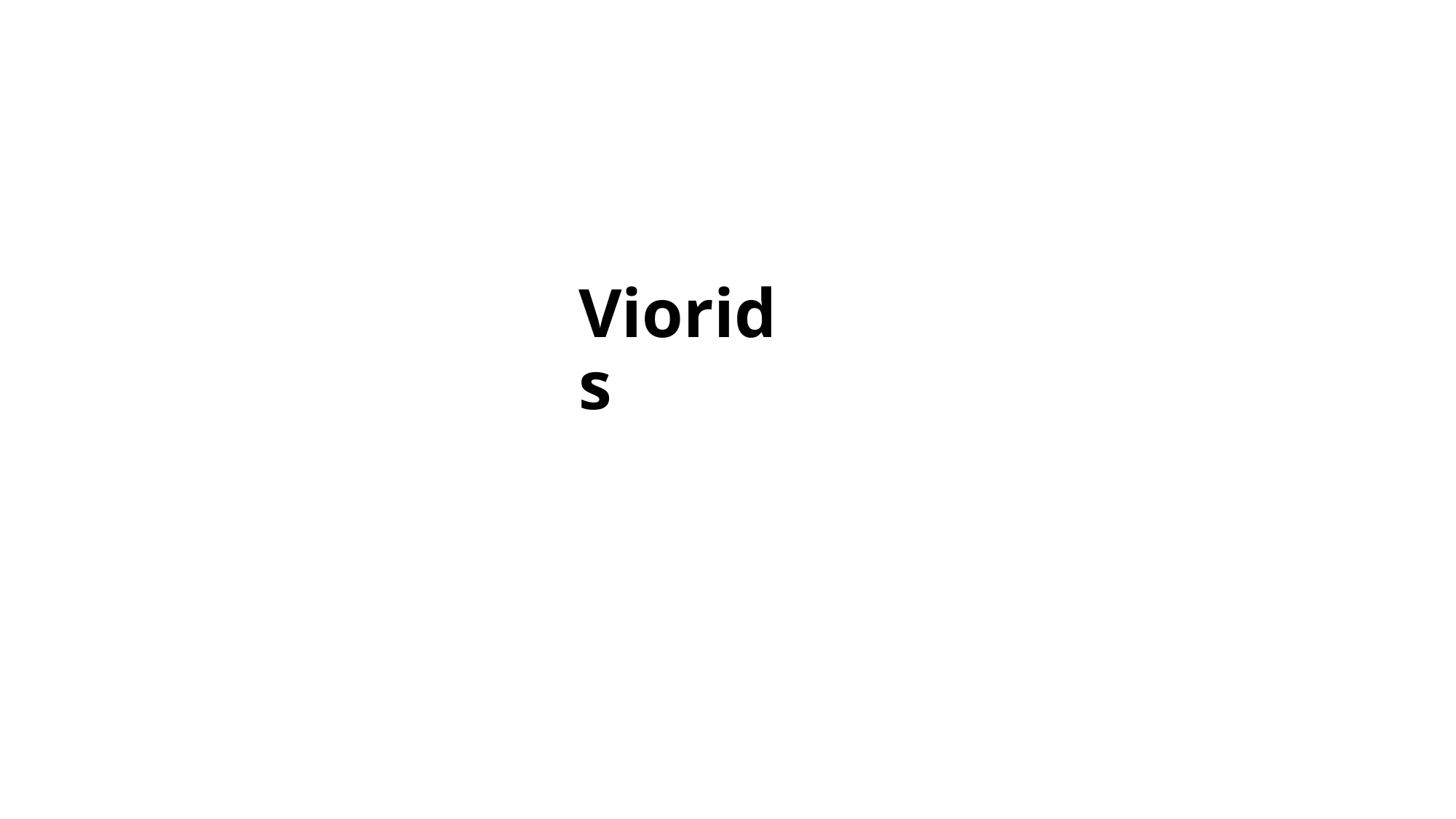

# Viorids

## Slide 11
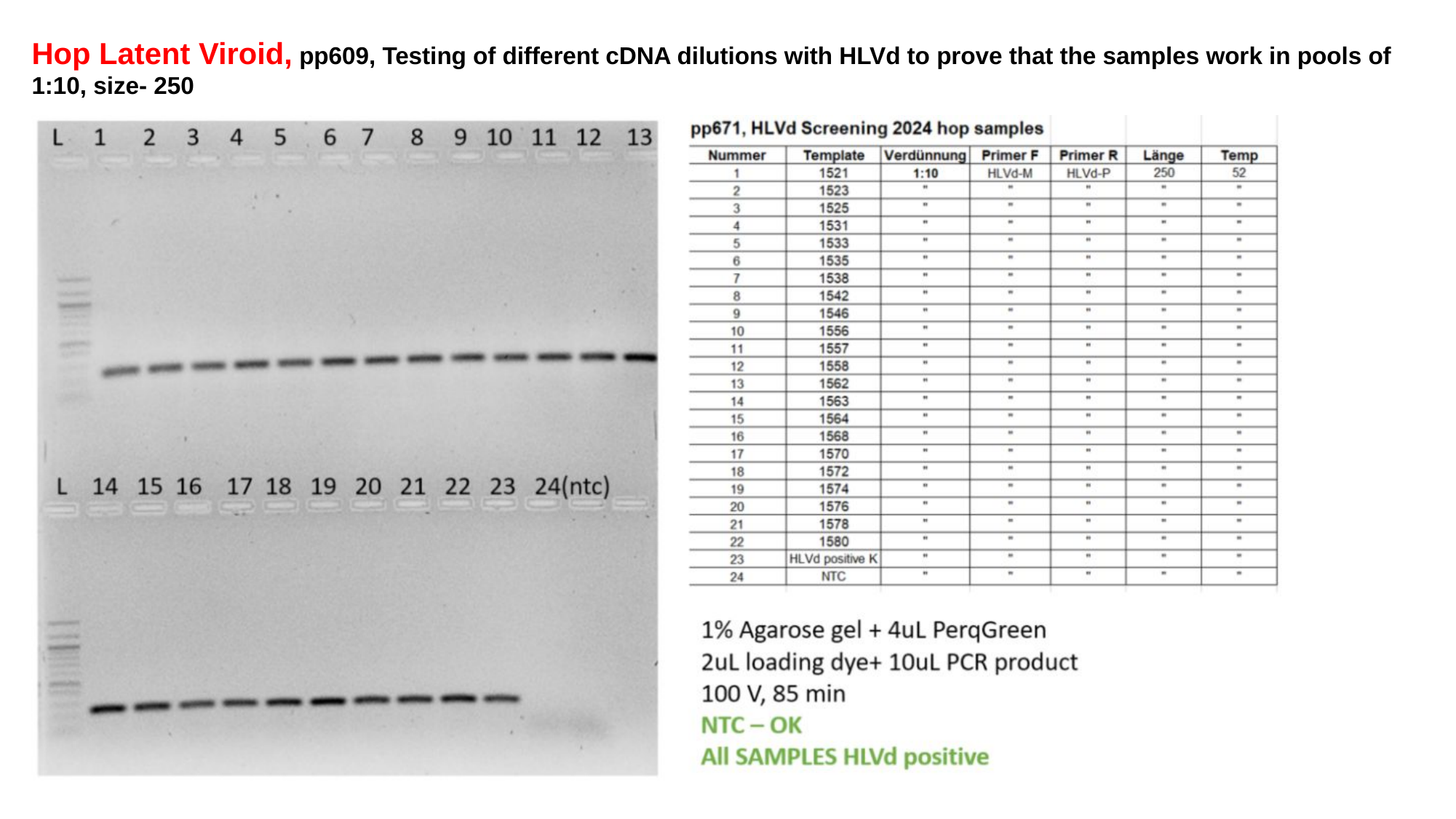

Hop Latent Viroid, pp609, Testing of different cDNA dilutions with HLVd to prove that the samples work in pools of 1:10, size- 250

## Slide 12
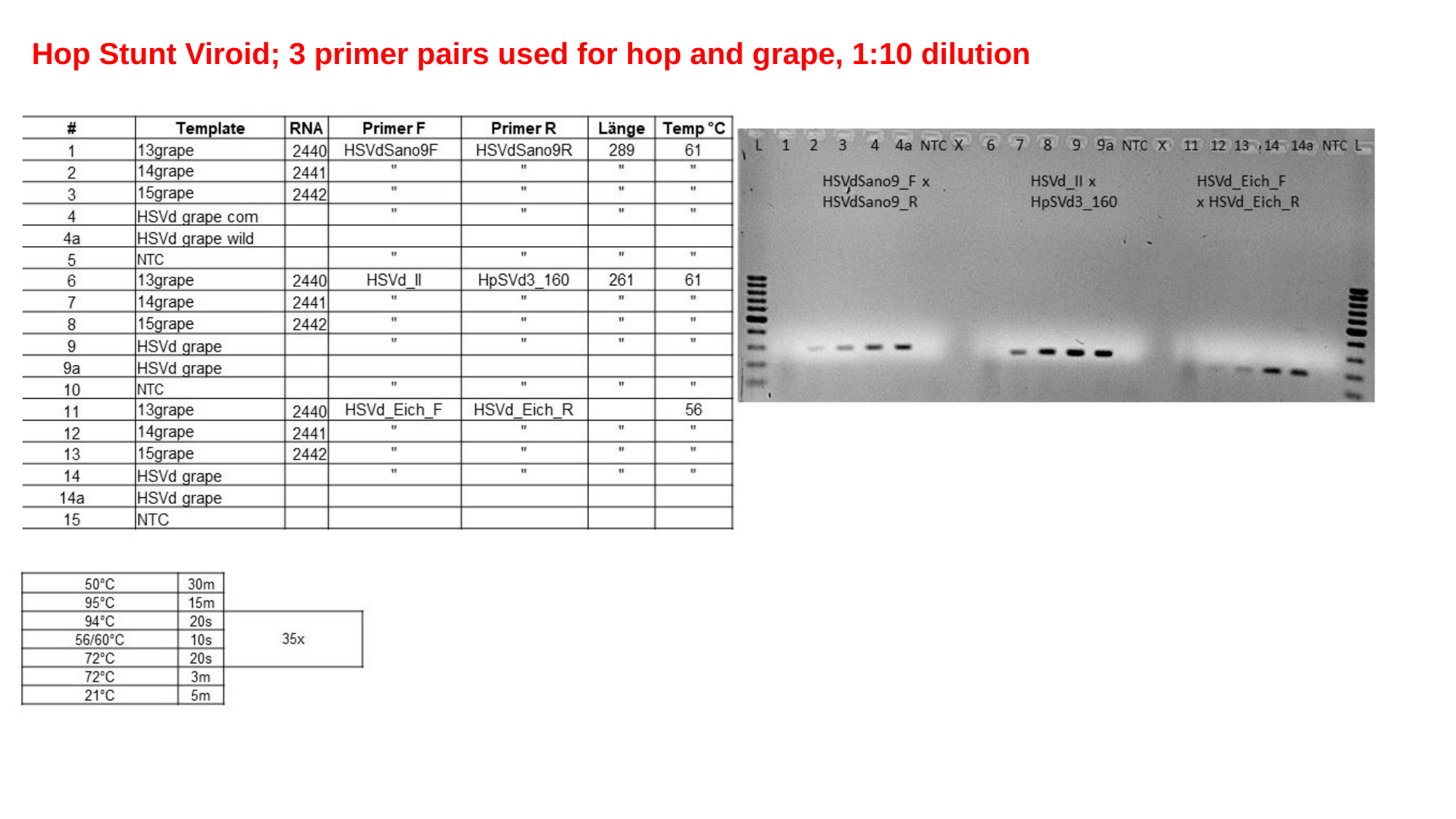

Hop Stunt Viroid; 3 primer pairs used for hop and grape, 1:10 dilution

## Slide 13
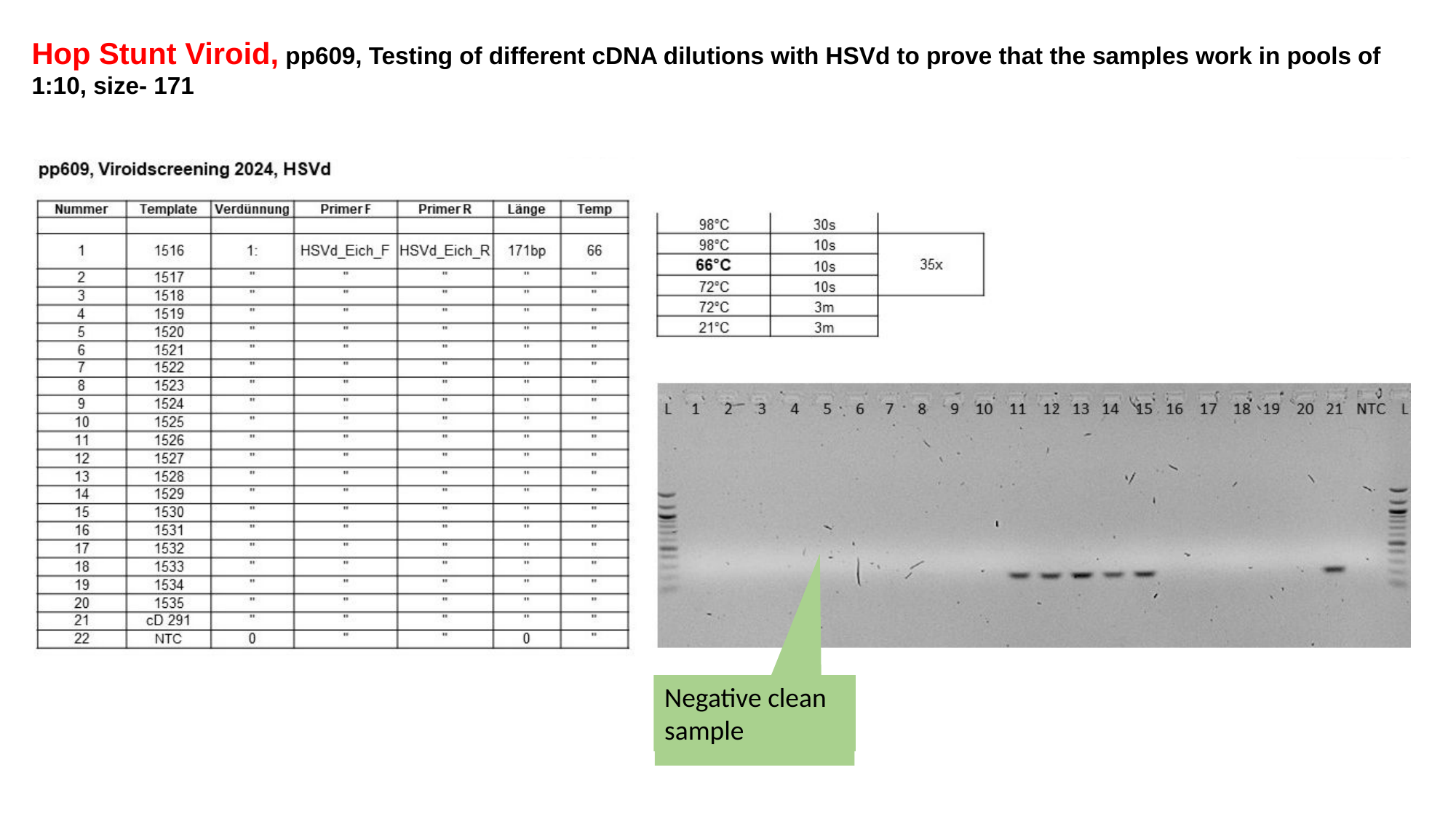

Hop Stunt Viroid, pp609, Testing of different cDNA dilutions with HSVd to prove that the samples work in pools of 1:10, size- 171
Negative clean sample
